# Nutrients or resin? – The relationship between resin and food foraging in stingless bees

**DOI:** 10.1002/ece3.10879

**Published:** 2024-02-08

**Authors:** Gemma Nydia Villagómez, Alexander Keller, Claus Rasmussen, Pablo Lozano, David A. Donoso, Nico Blüthgen, Sara Diana Leonhardt

**Affiliations:** ^1^ Department of Animal Ecology and Tropical Biology University of Würzburg, Biocenter Würzburg Germany; ^2^ Faculty of Biology, Cellular and Organismic Networks LMU Munich Planegg‐Martinsried Germany; ^3^ Department of Agroecology Aarhus University Slagelse Denmark; ^4^ Universidad Estatal Amazónica Puyo Ecuador; ^5^ Grupo de Investigación en Ecología y Evolución en los Trópicos‐EETROP Universidad de Las Américas Quito Ecuador; ^6^ Department of Biology, Ecological Networks Technical University of Darmstadt Darmstadt Germany; ^7^ TUM School of Life Sciences, Plant‐Insect‐Interactions Technical University of Munich Freising Germany

**Keywords:** DNA metabarcoding, interaction networks, Meliponini, nectar, resource collection

## Abstract

Stingless bees are important pollinators in tropical forests. Yet, we know little about their foraging behavior (e.g., their nutritional requirements or their floral sources visited for resource collection). Many stingless bees not only depend vitally on pollen and nectar for food but also on resin for nest building and/or defense. However, it is unclear whether the large effort devoted to collecting resin as a non‐food resource by certain stingless bees affects their foraging behavior. Therefore, in this study, we analyzed differences in foraging patterns (i.e., foraging activity, proportion of collected resources, and specialization in plants visited) and resource nutritional composition (i.e., sucrose amount in nectar and amino acids in pollen) of seven different stingless bee species (eleven wild colonies) in north‐western Ecuador with a particular focus on the role of resin collection. We found that species with a high resin intake tended to be more active than species with a low resin intake. The foragers per minute invested for pollen collection were similar across all species. Sucrose intake per minute differed between some species but was not affected by increased resin intake. Interestingly, high and low resin collectors partly differed in the plants visited for pollen collection. Pollen amino acid profiles largely, but not completely, overlapped between the two resin collection groups. Our findings show that the foraging patterns and plant choices of stingless bees may vary depending on their resin intake, highlighting the need for more research focusing on resin collection and use by stingless bees.

## Graphical abstract

We analyzed the role of resin intake on the foraging behavior and resource intake of tropical stingless bees in Ecuador. We show that stingless bee species with a large workforce devoted to collecting resin were overall more active, and foraged on pollen from slightly different spectra of plants, suggesting that species engaging in high resin collection might compensate for their comparatively low number of food foragers by increasing overall foraging activity and exploring specific plants for resource allocation.

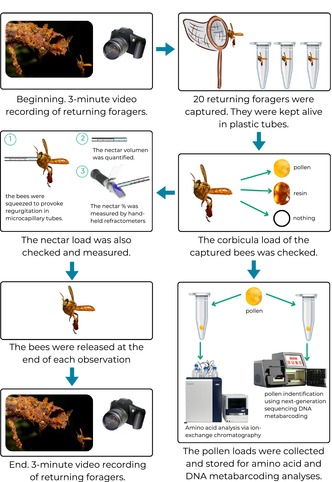

## Resumen

Las abejas sin aguijón son polinizadores importantes en los bosques tropicales. Sin embargo, sabemos poco acerca de su comportamiento de forrajeo (e.g., sus requisitos nutricionales o las fuentes florales visitadas para la recolección de recursos). Muchas abejas sin aguijón dependen vitalmente no solo de polen y de néctar como alimento, sino también de resinas para la construcción de su nido y/o defensa. Sin embargo, no está claro si el gran esfuerzo dedicado a la recolección de resina como recurso no alimentario de ciertas abejas sin aguijón afecta su comportamiento de forrajeo. Por lo tanto, en este estudio, analizamos las diferencias en los patrones de forrajeo (i.e., actividad de forrajeo, proporción de recursos recolectados y especialización en las plantas visitadas) y la composición nutricional de los recursos recolectados (i.e., cantidad de sacarosa en el néctar y de aminoácidos en el polen) de siete especies diferentes de abejas sin aguijón (once colonias silvestres) en el noroeste de Ecuador, con un enfoque particular en el rol de la recolección de resina. Encontramos que las especies con una recolección alta de resina tienden a ser más activas que las especies con una recolección baja de resina. La cantidad de forrajeadores por minuto dedicada a la recolección de polen fue similar en todas las especies. La ingesta de sacarosa por minuto difirió entre algunas especies, pero no se vio afectada por un aumento en la recolección de resina. Interesantemente, las abejas con una recolección alta y baja de resina difirieron parcialmente en las plantas que visitaron para la recolección de polen. Entre los dos grupos de recolección de resina también hubo diferencias con respecto al perfil de aminoácidos en el polen que recolectaron. El perfil de aminoácidos se sobrelapaba, pero no completamente, entre los dos grupos. Nuestros resultados muestran que los patrones de forrajeo y las elecciones de plantas de las abejas sin aguijón pueden variar según su consumo de resina, destacando la necesidad de hacer más investigaciones centradas en la recolección y el uso de resina por parte de las abejas sin aguijón.

## 1 INTRODUCTION

Stingless bees are essential pollinators in tropical forests (e.g., Michener, 2007; Roubik, 1989). Despite their importance, we know little about their foraging patterns, such as their collected resources or the floral sources visited for obtaining those resources. Like most bee species, pollen and nectar are vital to them and their larvae (Michener, 2007). Pollen is rich in proteins and lipids (Roulston & Cane, 2000), while nectar is rich in carbohydrates (mostly sugars) (Nicolson & Thornburg, 2007). Stingless bees have species‐specific preferences for sugar concentrations ranging from 20 to 70% (Biesmeijer & Ermers, 1999; Leonhardt et al., 2007; Roubik & Buchmann, 1984). Concerning pollen, stingless bees are broadly polylectic; that is, they collect pollen from a wide range of plant species (Biesmeijer & Slaa, 2006; Ferreira et al., 2021; Oliveira et al., 2017; Vossler, 2018), with certain differences in preference between species (Vossler, 2018).

Besides nectar and pollen, stingless bees also collect other resources (Roubik, 1989). For many stingless bee species, plant resins play a vital role (e.g., Leonhardt, 2017). They are not only waterproof and sticky, but also often fungicidal, bactericidal, and bacteriostatic, making them the ideal material for construction and defense (Armbruster, 1984; Chui et al., 2022; Greco et al., 2010; Leonhardt & Blüthgen, 2009; Roubik, 2006). Moreover, resin compounds enrich the chemical diversity of the cuticular profile of some species, which can modulate their aggression toward other species (Leonhardt, 2017).

Species differ strongly in the amount and proportion of resin foragers (e.g., Layek & Karmakar, 2018; Leonhardt et al., 2007, 2014; Leonhardt & Blüthgen, 2009; Wallace & Lee, 2010). Levels of resin use vary depending on predator pressure (Leonhardt & Blüthgen, 2009), colony developmental stage, and species characteristics, such as nest building (i.e., the amount of resin mixed with wax to build nest structures) and defense strategies (e.g., coating nest entrance with sticky resin layer or directly applying resin to predators, physical defense, such as biting, or hiding and closing the nest entrance) (Shanahan & Spivak, 2021) (see Table 1 for our study species). However, the factors influencing variation in the amount of resin collection and use as well as its effect on colony dynamics are still unclear. For example, for those species that use large amounts of resin for nest construction or defense, there could be a trade‐off between collecting resin and collecting food resources as pollen cannot be mixed with resin (Armbruster, 1984). In fact, a large foraging effort assigned to resin as a non‐food resource may require some sort of compensation, for example, by increasing overall activity or collecting food resources from different plant species with different nutritional quality (e.g., high protein or sucrose content).

**TABLE 1:** Colony name (ID) of stingless bee species found in the natural reserves Río Canandé and Tesoro Escondido.

Colony ID	Species	Reserve	Defense strategy (ref.)	Foraging strategy (ref.)	Nest entry material (ref.)	Resin in cuticular profile (yes/no), collection classification (high/low), and % ± *SD* resin foragers
BN9	*Nannotrigona tristella*	Canandé	Hide and seal nest entry with wax (Rasmussen & Gonzalez, 2017)	Within the genus: solitary foragers that recruit (Jarau et al., 2003)	Wax (Rasmussen & Gonzalez, 2017)	No, low (4.51 ± 7.26)
BN8	*Plebeia* sp.	Canandé	Diverse genus. Some species hide or are aggressive (Couvillon et al., 2008)	Diverse genus. Some species have solitary foragers that recruit (Aguilar et al., 2005; Slaa et al., 2003)	Diverse genus. Some species make the nest entry only with wax, others put resin around nest entrance (Wittmann, 1989)	No, low (6 ± 5.48)
BN1	*Ptilotrigona occidentalis*	Canandé	Aggressive (Camargo & Pedro, 2004)	No information	Resin around nest entrance (Camargo & Pedro, 2004)	Yes, high (25.11 ± 4.63)
BN4	*Scaptotrigona* sp. 1	Canandé	Aggressive (Couvillon et al., 2008; Jungnickel et al., 2004)	Within the genus: group foragers that recruit (Jarau et al., 2003; Sánchez et al., 2004; Slaa et al., 1997)	Wax (C. Rasmussen *pers. obs*.)	No, low (5.06 ± 5.03)
BT5	*Scaptotrigona* sp. 1	Tesoro Escondido	Aggressive (Couvillon et al., 2008; Jungnickel et al., 2004)	Within the genus: group foragers that recruit (Jarau et al., 2003; Sánchez et al., 2004; Slaa et al., 1997)	Wax (C. Rasmussen *pers. obs*.)	No, low (2.20 ± 3.06)
BN2	*Scaptotrigona* sp. 2	Canandé	Aggressive (Couvillon et al., 2008; Jungnickel et al., 2004)	Within the genus: group foragers that recruit (Jarau et al., 2003; Sánchez et al., 2004; Slaa et al., 1997)	Wax (C. Rasmussen *pers. obs*.)	No, low (1 ± 2.24)
BT1	*Tetragona ziegleri*	Tesoro Escondido	No information	Solitary. Poor recruiters (Slaa et al., 2003)	Resin (Roubik, 1990)	Yes, high (39.89 ± 13.18)
BT7	*Tetragona ziegleri*	Tesoro Escondido	No information	Solitary. Poor recruiters (Slaa et al., 2003)	Resin (Roubik, 1990)	Yes, high (44.78 ± 7.28)
BN3	*Tetragonisca angustula*	Canandé	Apply resin to predators (Wittmann, 1985)	Solitary. Poor recruiters (Aguilar et al., 2005)	Resin around nest entrance (C. Rasmussen *pers. obs*.)	Yes, high (42.19 ± 14.84)
BN5	*Tetragonisca angustula*	Canandé	Apply resin to predators (Wittmann, 1985)	Solitary. Poor recruiters (Aguilar et al., 2005)	Resin around nest entrance (C. Rasmussen *pers. obs*.)	Yes, high (42.53 ± 21.95)
BN7	*Tetragonisca angustula*	Canandé	Apply resin to predators (Wittmann, 1985)	Solitary. Poor recruiters (Aguilar et al., 2005)	Resin around nest entrance (C. Rasmussen *pers. obs*.)	Yes, high (57.81 ± 18.18)

*Note*: The table includes information on: (a) the colony and species name, (b) the reserves where the colonies were located, (c) defense and foraging strategy of each species, (d) whether the nest entry is made solely out of wax or whether resin is used (with respective references), (e) whether they incorporate resin compounds into their cuticular profile (cp), (f) resin foraging behavior (mean percentage (%) ± SD of foragers returning with resin, obtained with the data of the 5 days observation), and (g) resin collection classification: low (≤20% resin foragers and absence of resin‐derived compounds in their cuticular profiles) or high resin collectors (>20% resin foragers and presence of resin‐derived compounds in their cuticular profiles).

In this study, we explored differences in foraging patterns (returning foragers/minute, proportion of collected resources, and specialization in pollen sources, that is, plants and plant life forms visited) and resource nutritional composition (sucrose amount in nectar and amino acids profiles of pollen) of 11 stingless bee nests representing seven species, with particular focus on the role of resin collection. We expected differences in foraging activity among species as documented by Leonhardt et al. (2007). Additionally, we anticipated that bees with higher foraging activity (measured as foragers per minute) would also exhibit higher sucrose intake per minute, as observed for *Tetragonula carbonaria*, (Leonhardt et al., 2014). We also expected that stingless bees show little specialization in pollen collection, but have preferences for specific plant life forms, as stingless bees are broadly polylectic, but forage preferentially in either the understory or canopy (Nagamitsu et al., 1999). Finally, we also expected that they collect pollen with similar amino acid profiles as seen for bumblebees (Kriesell et al., 2017).

To understand the effect of resin collection on the bees' foraging behavior, we classified our study species as low and high resin collectors (see Section 2, Table 1). We hypothesized that high resin collectors show an overall higher foraging activity and collect pollen of higher protein content and nectar of higher sucrose content compared to low resin collectors to compensate for the workforce (i.e., foragers/minute coming back with one specific resource) invested in the allocation of a non‐food source. Finally, we investigated whether the high resin collectors collected pollen from plants that also produce resin (in different foraging trips), as this might save the colony search efforts.

## 2 METHODS

### 2.1 Study region

Observations took place in the natural reserves Río Canandé (00°31.576′ N, 079°12.771′ W) and Tesoro Escondido (00°32.507′ N, 079°08.702′ W), in northwestern Ecuador. Both reserves are in the lowland forest (between 100 and 500 m.a.s.l.) of the biogeographical region Chocó‐Darién which has annual precipitation between 3000 and 5000 mm (Ministerio del Ambiente, 2011) and harbors many endemic species (Lozano et al., 2022; Myers et al., 2000).

### 2.2 Bee sampling and foraging observations

**FIGURE 1:**
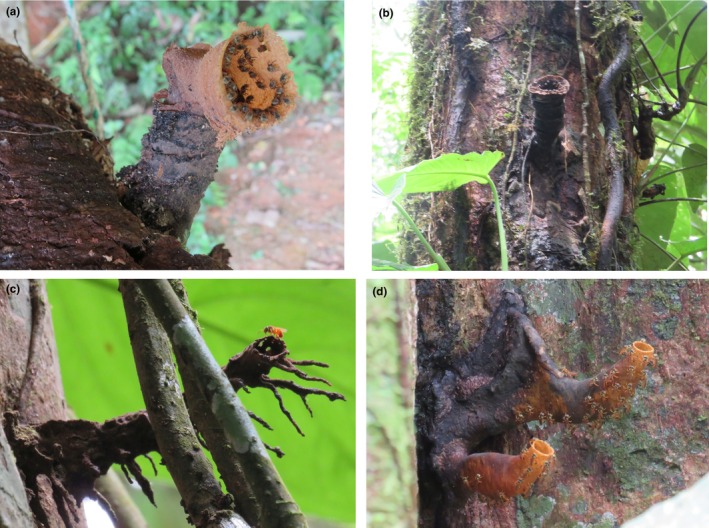
Stingless bee colonies studied. BN9 *Nannotrigona tristella* (a), BN4 *Scaptotrigona* sp. 1 (b), BT7 *Tetragona ziegleri* (c), and BN5 *Tetragonisca angustula* (d).

Eleven wild stingless bee colonies were located by walking through the forest (Figure 1, Table 1). For each colony, we sampled specimen bees for identification by CR and DNA barcoding using the BOLD Identification System (Ratnasingham & Hebert, 2007) (Appendix S1). The collection and export permits (MAE‐DNB‐CM‐2015‐0068, 144‐2019‐EXP‐CM‐FAU‐DNB/MA, respectively) were issued by the Ministerio del Ambiente from Ecuador. Voucher specimens were deposited in the museum collection at the Escuela Politécnica Nacional in Quito.

The located colonies correspond to seven species: 1 nest from *Nannotrigona tristella* Cockerell, 1922, 1 *Plebeia* sp., 1 *Ptilotrigona occidentalis* Schulz, 1904, 2 *Scaptotrigona* sp. 1 (belonging to the taxonomical group B established by Engel (2022)), 1 *Scaptotrigona* sp. 2 (taxonomical group A (Engel, 2022). DNA barcoding indicates close relation to specimens identified as *Scaptotrigona pectoralis* (BOLD Sequence ID: ASINH786‐12.COI‐5P) with 99.68% sequence identity), 2 *Tetragona ziegleri* Friese, 1900, and 3 *Tetragonisca angustula* Latreille, 1811.

We classified our studied species into two groups (low and high resin collectors) based on their resin collection behavior (species with ≤20% resin foragers or with >20%, respectively, Table 1), and on the absence/presence (respectively) of resin‐derived compounds in their cuticular profiles (using information from Leonhardt et al. (2013), Drescher et al. (2019), and analyzing their cuticular profiles (Figure S1, Appendix S1)).

Each nest was visited between February and April 2019 (rainy season) on five non‐consecutive and non‐rainy days to randomize data collection across the entire study period and rule out effects of climatic variation over the day. Note that we did not collect environmental data. The observations were done at specific points of time during the day (between 8 am and 5 pm) and lasted around 2 h per colony with points of times randomized over subsequent visits. Occasionally, in Tesoro Escondido reserve, nests were visited on the consecutive day, but at a different time than the previous day.

**FIGURE 2:**
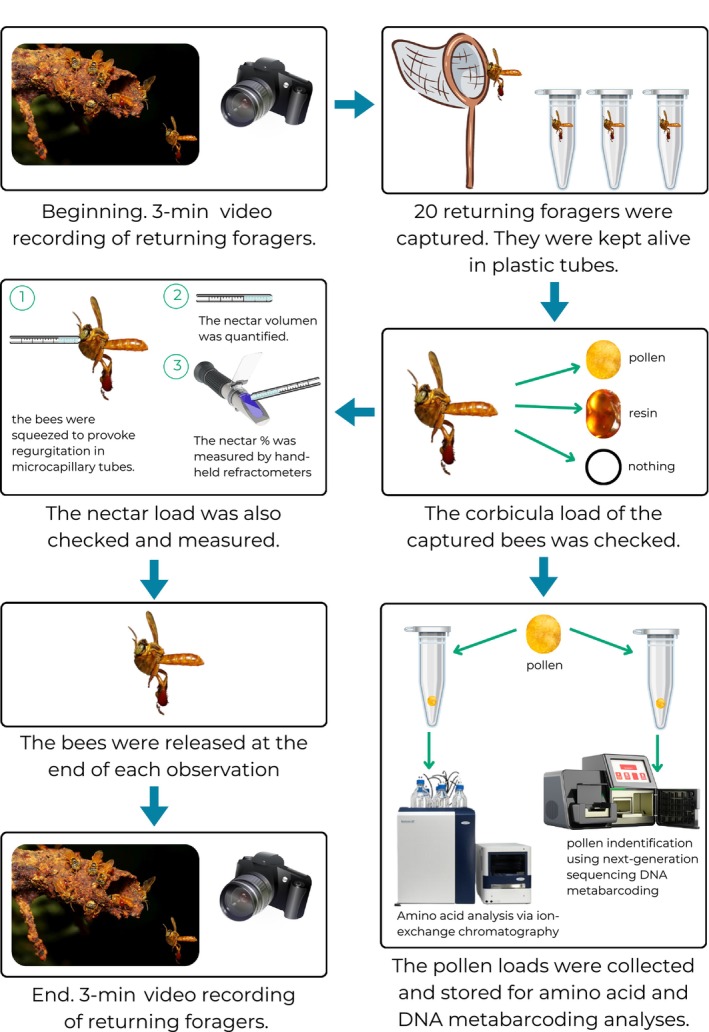
Employed methods for bee sampling and foraging observations. At the beginning of the observation period, returning foragers were recorded for 3 min to measure foraging activity. Afterwards, 20 different returning foragers (10 in the case of *Plebia* sp., as there were only very few active foragers) were captured and their corbicula and nectar loads were recorded. We used a 5 μL microcapillary tube to measure the sugar concentrations of nectar loads by placing the tube near the forager's mouth and carefully squeezing its abdomen. The volume of the regurgitated nectar was quantified, and its concentration (in percentage) was measured using hand‐held refractometers. The pollen loads carried on each leg were collected in different vials, frozen, and stored for amino acid and DNA metabarcoding analyses. Finally, at the end of the observation period, all returning foragers were again recorded for 3 min. Bee photo by Philipp Hoenle.

Each day, 20 different returning foragers (10 in the case of *Plebia* sp., due to few foragers) were captured using an insect net, and their corbicula and nectar load were visually checked (Figure 2). Loads of each bee were categorized into no load (*nl*), only nectar (including all fluids regurgitated from the crop) (*n*), only pollen (*p*), only resin (including all indistinguishable sticky substances) (*r*), pollen and nectar (*pn*), resin and nectar (*rn*), or resin and pollen (*rp*). In doing so, we could determine how much effort was allocated to each resource. Captured bees were kept alive in plastic tubes, with holes in the lid to ensure ventilation, and released at the end of each observation period to avoid recapturing the same individual.

Each day, we video‐recorded (camera Canon SX540 HS) the number of returning foragers for 3 min before and after capture (Figure 2). The average number of returning foragers/minute (i.e., activity, *A*) was calculated for each day and colony. To account for probable differences in colony size, daily activity was normalized (*A1*) using the maximum number of returning foragers/minute for each colony (max; *A*1 = *A*/max). We then calculated the number of foragers/minute with the load categories mentioned above (*Fm*_(*nl*,*n*,*p*,*r*,*pn*,*rn*,*rp*)_) following Leonhardt et al. (2014):

$${\mathrm{Fm}}_{\left(\mathrm{nl},n,p,r,\mathrm{pn},\mathrm{rn},\mathrm{rp}\right)}=A1*P_{\left(\mathrm{nl},n,p,r,\mathrm{pn},\mathrm{rn},\mathrm{rp}\right),}$$

where *P*_(*nl*,*n*,*p*,*r*,*pn*,*rn*,*rp*)_ is the proportion of foragers that carried a particular resource.

All captured bees were carefully squeezed to provoke regurgitation. The volume of regurgitated nectar was quantified in 5 μL microcapillary tubes, and its concentration (*c*, in percentage) was measured to the nearest 0.5 g/g sucrose equivalent by hand‐held refractometers (Eclipse 45–81 (0–50 °Brix) and Eclipse 45–82 (45–80 °Brix), Bellingham + Stanley) (Figure 2). The sugar percentage was converted into *x* (μg/μL) following Leonhardt et al. (2014):

$$x=-0.0928+10.0131*c+0.0363*c^{2}+0.0002*c^{3}$$

The sucrose amount (*ms*, in mg) was obtained by multiplying *x* by the nectar volume and transformed into milligrams. We finally calculated the sugar intake (mg/min) (*S*_*in*_) as follows:

$$S_{\text{in}}=\mathrm{ms}*A1*P_{\left(\mathrm{Pn}+\mathrm{Ppn}+\mathrm{Prn}\right),}$$

where *P*_(*Pn+Ppn+Prn*)_ is the sum of the proportion of all foragers returning with nectar.

The pollen loads carried on each leg were collected in different vials, frozen, and stored for chemical and DNA metabarcoding analyses (Figure 2). For DNA metabarcoding, pollen pellets were sequenced individually. For the chemical analysis, pollen pellets from all pollen foragers collected for one colony at 1 day were pooled to obtain sufficient material for the analysis. Additional pollen foragers were captured for colonies BN1, BN2, BN3, BN4, and BN8.

### 2.3 Pollen metabarcoding and plant assessment

We applied next‐generation sequencing pollen DNA metabarcoding to construct bee–plant interaction networks (Bell et al., 2016, 2017; Keller et al., 2015; Pornon et al., 2017). DNA metabarcoding of the ITS2 plant region was performed following Sickel et al. (2015) with hands‐on details provided in Campos et al. (2021), Ankenbrand et al. (2015), Edgar and Flyvbjerg (2015), Edgar (2016a, 2016b), Elliot et al. (2021), and in the Appendix S1. Sequencing reads were quality filtered, denoised to amplicon sequence variants (ASVs), chimera filtered, and taxonomically classified with VSEARCH (Rognes et al., 2016) against custom reference databases for the study region created with the BCdatabaser (Keller et al., 2020). Read counts per sample were transformed to relative read abundances (RRAs) by dividing the number of reads per taxon and samples by the sample sum. We excluded samples with <100 reads and the taxa accounting for <1% of reads per sample to determine the most abundant plant species visited (phyloseq R‐package, McMurdie & Holmes, 2013) (details in Appendix S1).

Afterwards, we also determined the life forms (i.e., epiphyte, herb, liana, shrub, tree, and tree or shrub) of the taxa accounting for more than 10% of reads per sample (after the 1% filter) and if they are known to produce resin based on taxonomic expertise of PL, Gentry (1996), the Global Biodiversity Information Facility (GBIF) (GBIF.org, 2020), and the Catalog of the Vascular Plants of Ecuador (internet version) (Missouri Botanical Garden, 1999). For resin production, additional literature was consulted (Table S1). Note we could not identify the life form of some of the plants, which we then classified as “non determined”.

### 2.4 Analysis of pollen amino acids

Pollen protein‐bound amino acids were analyzed by ion‐exchange liquid chromatography (IEC, Biochrom 20 Plus Amino Acid Analyzer) following Leonhardt and Blüthgen (2012) (Appendix S1). We calculated the proportion of each amino acid and the total concentration of pollen amino acids (μg/mg of pollen, wet weight) in each sample.

### 2.5 Statistical analysis

All analyses were performed with the software R version 4.2.1 (R Core Team, 2022). For all linear, linear mixed‐effect models (lme4 R‐package; Bates et al., 2015), generalized linear (glm), generalized linear mixed models, and generalized linear mixed models using template model builder (glmmTMB, glmmTMB package, Brooks et al., 2017), model diagnostics were done with the DHARMa package (Hartig, 2021). For glms with quasibinomial distribution, the residual diagnosis was visually done using residuals vs. fitted values, and quantile–quantile plots (car package, Fox & Weisberg, 2019). Pairwise comparisons were assessed by computing contrasts of estimated marginal means (adjustment method: Holm; emmeans package; Lenth, 2018).

#### 2.5.1 Differences in foraging patterns, resource intake, and nectar collection

We tested for differences in (a) foraging activity (*A1*) (the maximum values, i.e., *A1* = 1, were excluded), and (b, c) proportion of returning foragers and foragers/minute with no load, only nectar, only pollen, only resin, pollen‐nectar (*T. angustula* was excluded from this category as no forager came with these two resources together), resin‐nectar (*N. tristella* and *Plebeia* sp. were excluded here for the same reason), and total pollen (*P*_*p*_ *+ P*_*pn*_ *+ P*_*rp*_/*Fm*_*p*_ *+ Fm*_*pn*_ *+ Fm*_*rp*_) between species. The category resin‐pollen was excluded as very few foragers across species and colonies returned with both resources simultaneously. Lastly, we analyzed differences in (d) nectar sucrose amount and intake/minute, and (e) pollen amino acid content (see Table 2 for the implemented statistical models).

**TABLE 2:** Implemented statistical models for analyzing species differences and differences between resin collection categories (explanatory variables) in the studied variables (response variables).

Explanatory variable	Response variable	Data transformation	Implemented models	*p*‐value obtention
Species	Foraging activity	None	lm	*F*‐test (function lm, stats package)
	Proportion of foragers with each resource	None	glm (quasibinomial distribution)	*F*‐test (function ANOVA, type II, car package)
	Foragers/minute with only resin, pollen‐nectar, resin‐nectar	Arcsine transformation	lm	*F*‐test (function lm, stats package)
	Foragers/minute with no load, only nectar, only pollen, total pollen	None	glmmTMB (log‐linked hurdle‐gamma distribution (zi Gamma))	Wald *χ* ^*2*^ test (function ANOVA, type II)
	Sucrose amount Sucrose intake/minute	None	glmmTMB (log‐linked gamma distribution. Species as dispersion parameter)	Wald *χ* ^*2*^ test (function ANOVA, type II)
	Proportion of visited: herbs, shrubs, trees	None	glm (binomial distribution)	Likelihood‐ratio tests (function ANOVA, type II)
	Total concentration of pollen amino acids	None	lm	*F*‐test (function lm, stats package)
Resin collection	Foraging activity	None	lmm	*F*‐test (function ANOVA, type II, lmerTest package, Kuznetsova et al., 2017)
	Foragers/minute with only resin	Arcsine transformation	lmm	*F*‐test (function ANOVA, type II)
	Foragers/minute with no load, only nectar, only pollen, pollen‐nectar, resin‐nectar, total pollen	None	glmmTMB (zi Gamma distribution)	Wald *χ* ^*2*^ test (function ANOVA, type II)
	Sucrose amount Sucrose intake/minute	None	glmmTMB (log‐linked gamma distribution)	Wald *χ* ^*2*^ test (function ANOVA, type II)
	Proportion of visited: herbs, shrubs, trees, and resin‐producing plants	None	glmm	Wald *χ* ^2^‐tests (function ANOVA type II)
	Total concentration of pollen amino acids	None	lmm	*F*‐test (function ANOVA)

*Note*: It is also indicated whether the data were transformed, and the tests used to obtain the *p*‐values of the models. The functions and the R‐packages (package citation is provided, when necessary) used to implement those tests are also given. Model's abbreviations: lm (linear models), glm (generalized linear models), lmm (linear mixed‐effect models), glmm (generalized linear mixed models), and glmmTMB (generalized linear mixed models using template model builder).

To obtain valid models in some cases, we used glmmTMB with a log‐linked hurdle‐gamma distribution (zi Gamma) (Table 2), which overcomes the restriction of the classical gamma distribution that does not allow zero as a response (Brooks et al., 2017). Additionally, in two models “species” were used as dispersion parameters to account for heteroskedasticity (Table 2) (Brooks et al., 2017).

Using Akaike's information criterion (AIC) for model selection, we assessed if colony ID and (or) nest location (i.e., reserve) improved the explanatory force of our models and should therefore be included as random factors (details in Appendix S1: Statistical analysis). Models without random factor(s) had smaller AIC values (Table S2), indicating that intraspecific differences and nest location did not crucially affect model results and could, therefore, be excluded (Figure S2 shows an example of a similar percentage of foragers for each resource for different colonies of the same species).

#### 2.5.2 Differences in plant species visited and in pollen amino acid profiles

We used bee‐pollen‐based interaction networks to depict pollen sources used by the different bee species (Bosch et al., 2009). The network analysis was made using RRAs (following Peters et al., 2022). We calculated the quantitative network‐level specialization index, *H*_2_′, and the species‐level specialization index, *d*′ (Blüthgen et al., 2006) (bipartite R‐package; Dormann et al., 2008). *H*_2_′ and *d*′ range from 0 (in the case of *H*_2_′: the different bee species visit similar plants for pollen collection, and in the case of *d*′: the specific species' pollen hosts overlap with other species) to 1 (in the case of *H*_2_′: the different bees species visit different plants for pollen collection, and in the case of *d*′: the specific species' pollen hosts hardly overlap with other species). We used the null‐model approach to see if our obtained *H*_2_′ was significantly different from random networks (details in Appendix S1: Statistical analysis). For visualization, only the plant taxa accounting for more than 10% of reads were presented in the network.

Additionally, we analyzed differences in (a) the proportion of herbs, shrubs, and trees (life forms visited by almost all bee species) from which pollen was harvested, (b) the total concentration of pollen amino acids (Table 2), and (c) the proportional pollen amino acid profiles. For the latest, we conducted a classification analysis based on Breiman's random forest algorithm using species as the class predictor (package randomForest (Liaw & Wiener, 2002), 100,000 trees). With this analysis, we obtained the out‐of‐bag (OOB) estimate of error rate (which indicates the percentage of points that were misclassified in the training set) and the class errors for each species (which indicates grouping accuracy according to bee species: 0 indicates that all values were correctly classified and 1 that values could not be correctly classified).

#### 2.5.3 Interaction between food and resin foraging

##### Relationship between resin collection and general foraging patterns, resource intake, and nectar collection

To explore if intensive resin collection by high resin collectors might be compensated through increasing activity, we tested whether low and high resin collectors differed in (a) foraging activity and/or (b) the number of returning foragers/minute with each resource (see above). To assess if compensation might occur via intake of higher quality resources, we tested for differences in (c) sucrose amount and (d) sucrose intake/minute. For this, we used mixed‐effect models with species as a random factor (Table 2).

Additionally, for each species, we tested if the number of resin foragers/minute was correlated with the number of only pollen and only nectar foragers/minute using Spearman correlation tests (cor.test function, stats package).

To correct for multiple testing when using the same data set in the correlation tests and when using species and resin collection as explanatory variables, we adjusted *p*‐values using the Holm method (function: *p*.adjust, stats package). A significance level (α) of 0.05 was used for all models. The models' original *p*‐values are presented in the results and marked in bold if they were significant after adjustment.

##### Relationship between resin collection and visited plant species and pollen amino acid profiles

To analyze if resin collection correlated with specialization levels for pollen sources, we conducted a network analysis and calculated *H*_2_′ and *d*′ specialization indices for low and high resin collectors. As above, a null‐model approach was implemented. We additionally compared the proportion of herbs, shrubs, trees, and resin‐producing plants visited for pollen collection (Table 2).

We also tested for differences in the concentration of pollen amino acids (Table 2) and the proportional pollen amino acid profiles between the two groups. As above, we conducted a random forest analysis using resin collection as the classification predictor and examined which amino acids were most important for the classification (details in Appendix S1: Statistical analysis). A multidimensional scaling graph, using *1 – Random Forest proximities* as distances, was created for data visualization.

## 3 RESULTS

### 3.1 Differences in foraging patterns, resource intake, and nectar collection

**FIGURE 3:**
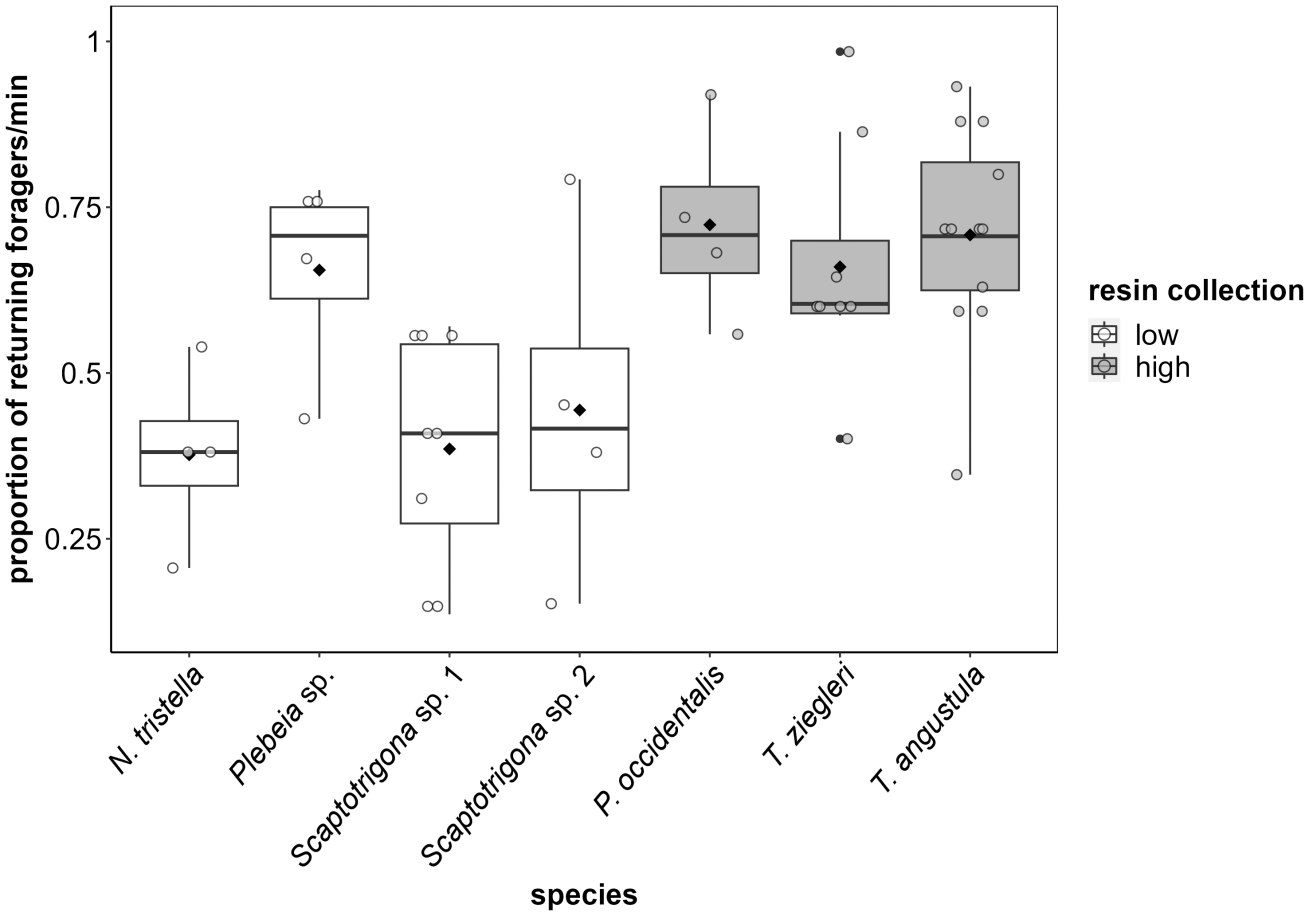
Flight activity (normalized based on the maximum number of returning foragers/minute for each colony) of the seven different stingless bee species observed in the forest of the reserves, grouped into low (white boxplots) and high (gray boxplots) resin collectors. Box plots display the median (thick bar), lower and upper quartile (boxes), and minimum and maximum values (whiskers) of the data set. The black diamonds represent the mean value of the data set. *n* = 4, except for *Scaptotrigona* sp. 1 and *T. ziegleri* with *n* = 8, and *T. angustula* with *n* = 12.

The seven stingless bee species differed in their foraging activity. In particular, *T. angustula* had almost double the number of foragers per minute compared with *N. tristella* and *Scaptotrigona* sp. 1 (lm: *F*_6,37_ = 4.9, *p* = .001, Figure 3, Table S3).

**FIGURE 4:**
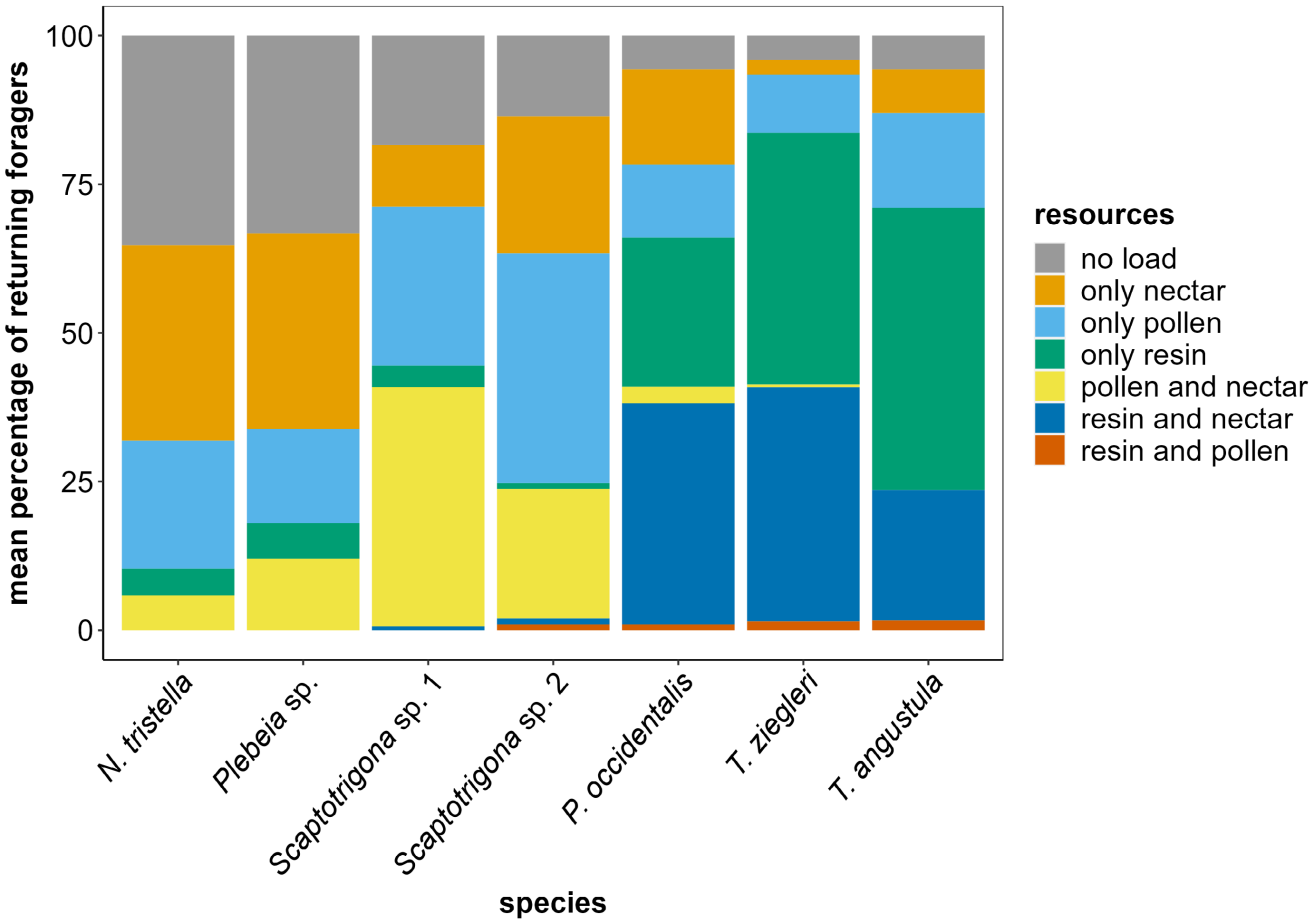
Mean percentage of returning foragers with no load, only nectar, only pollen, only resin, pollen and nectar, resin and nectar, and resin and pollen of the seven different stingless bee species observed in the forest of the reserves. Note the differences between the low resin collectors (*N. tristella*, *Plebeia* sp., *Scaptotrigona* sp. 1, *Scaptotrigona* sp. 2) and high resin collectors (*P. occidentalis*, *T. ziegleri*, and *T. angustula*) in the percentage of foragers coming back with resin.

The seven species also differed in their percentages of returning foragers with no load, nectar, pollen, resin, pollen‐nectar, resin‐nectar, and total pollen (Figure 4, Table 3), with no differences between colonies of the same species (Figure S2). The main differences between species were in the percentage of resin foragers (Table S4). For instance, up to 47% and 42% of *T. angustula* and *T. ziegleri*, respectively, and up to 25% of *P. occidentalis* foragers collected only resin, while only up to 1% of *Scaptotrigona* sp. 2, 4% of *N. tristella*, *Scaptotrigona* sp. 1, and 6% of *Plebeia* sp. foragers collected resin (Table 1, Figure 4, Table S4). Moreover, the total pollen percentage ranged from 12% (in *T. ziegleri*) to 67% (in *Scaptotrigona* sp. 1), and the percentage of foragers only returning with nectar ranged from 3% (in *T. ziegleri*) to 33% (in *N. tristella* and *Plebeia* sp.). Interestingly, *Ptilotrigona occidentalis*, *T. ziegleri*, and *T. angustula* had four times fewer foragers returning without any resources than *N. tristella* and *Plebeia* sp. (6, 4, and 6%, vs. 35% and 33%, respectively) (Figure 4).

**TABLE 3:** Differences between species and high and low resin collectors in the proportion of foragers and foragers per minute (calculated with the normalized foraging activity) with each resource.

	Species		Resin collection	
	*F/*χ^2^	*p*	*F/*χ^2^	*p*
**No load**				
Proportion	*F* = 9.4	**<.0001**	NA	NA
Foragers/min	*χ* ^2^ = 28.3	**<.0001**	*χ* ^2^ = 4.9	.03
**Only nectar**				
Proportion	*F* = 5.8	**.0001**	NA	NA
Foragers/min	*χ* ^2^ = 23.1	**.001**	*χ* ^2^ = 7.9	.005
**Only pollen**				
Proportion	*F* = 3.8	**.003**	NA	NA
Foragers/min	*χ* ^2^ = 6.3	.39	*χ* ^2^ = 0.09	.76
**Only resin**				
Proportion	*F* = 27.7	**<.0001**	NA	NA
Foragers/min	*F*_6,48_ = 26.6	**<.0001**	*F*_1,4.3_ = 71.3	**.0008**
**Pollen and nectar**				
Proportion	*F* = 14.2	**<.0001**	NA	NA
Foragers/min	*F*_5,34_ = 8.9	**<.0001**	*χ* ^2^ = 2.6	.11
**Resin and nectar**				
Proportion	*F* = 18.2	**<.0001**	NA	NA
Foragers/min	*F*_4,40_ = 18.7	**<.0001**	*χ* ^2^ = 33.3	**<.0001**
**Total pollen**				
Proportion	*F* = 13.9	**<.0001**	NA	NA
Foragers/min	*χ* ^2^ = 25.9	**.0002**	*χ* ^2^ = 7.4	.006

*Note*: *F*‐, *χ* ^2^ ‐, and *p*‐values are displayed. They were obtained with *F*‐ and Wald *χ* ^2^‐tests of the implemented generalized and linear models. Bold values indicate significant differences between species (*p* ≤ .05) after *p*‐value adjustment.

Results for foragers/minute with each resource were similar to the results for percentages (Table 3, Figure S3, Tables S3 and S4). For example, *P. occidentalis*, *T. ziegleri*, and *T. angustula* invested around five times more workforce into collecting resin than the other species. Interestingly and contrary to the percentage of only pollen foragers, all bee species invested a similar workforce in collecting only pollen (Table 3, Figure S3, Table S4). However, the overall pollen intake/minute was three to four times higher in *Scaptotrigona* sp. 1 than in *T. angustula* and *P. occidentalis*, or *T. ziegleri*, respectively (Figure S3).

**FIGURE 5:**
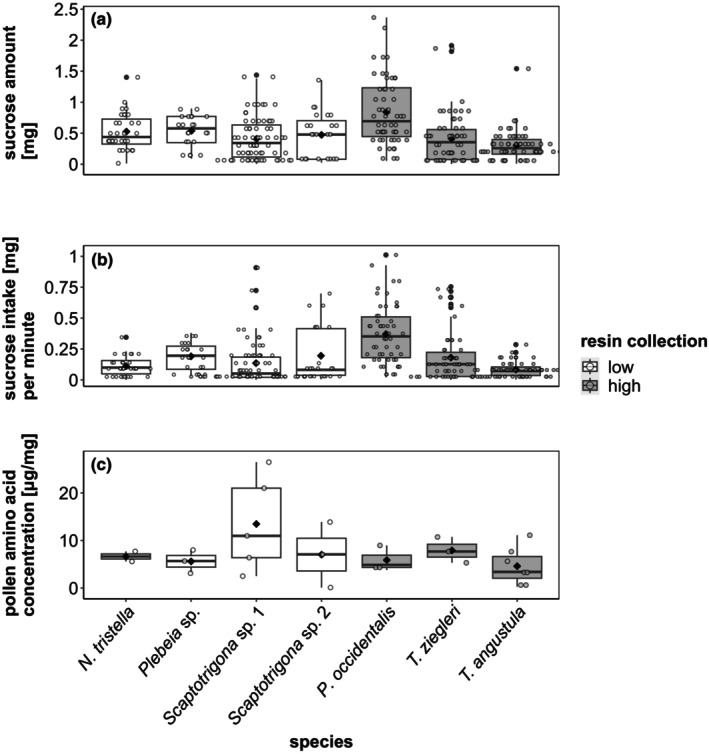
Sucrose amount in nectar collected (a), sucrose intake (mg) per minute (calculated based on the normalized flight activity) (b), and total pollen amino acid (μg/mg pollen) collected (c) by seven different stingless bee species observed in the forest of the reserves. Species are grouped into low (white boxplots) and high (gray boxplots) resin collectors. Box plots display the median (thick bar), lower and upper quartile (boxes), and minimum and maximum values (whiskers) of the data set. Black diamonds represent the mean value of the data set. In the case of sucrose amount and intake, *N. tristella* is represented with 31 nectar samples (*n*), *Plebeia* sp. *n* = 22, *P. occidentalis n* = 54, *Scaptotrigona* sp. 1 *n* = 69, *Scaptotrigona* sp. 2 *n* = 23, *T. ziegleri n* = 55, and *T. angustula n* = 58. In the case of pollen amino acids *n* = 3, except for *N. tristella* with *n* = 2, *Scaptotrigona* sp. 1 with *n* = 5, and *T. angustula* with *n* = 7.

Nectar sucrose content and sucrose intake/minute also differed between species (glmmTMB: *χ* ^2^ = 66.4, *p* < .001; *χ* ^2^ = 128.6, *p* < .001, respectively; Figure 5a,b, Table S5). For example, *Ptilotrigona occidentalis* had double sucrose intake/minute compared to most other species but did not significantly differ from *Scaptotrigona* sp. 2.

### 3.2 Differences in plant species visited and in pollen amino acid profiles

**FIGURE 6:**
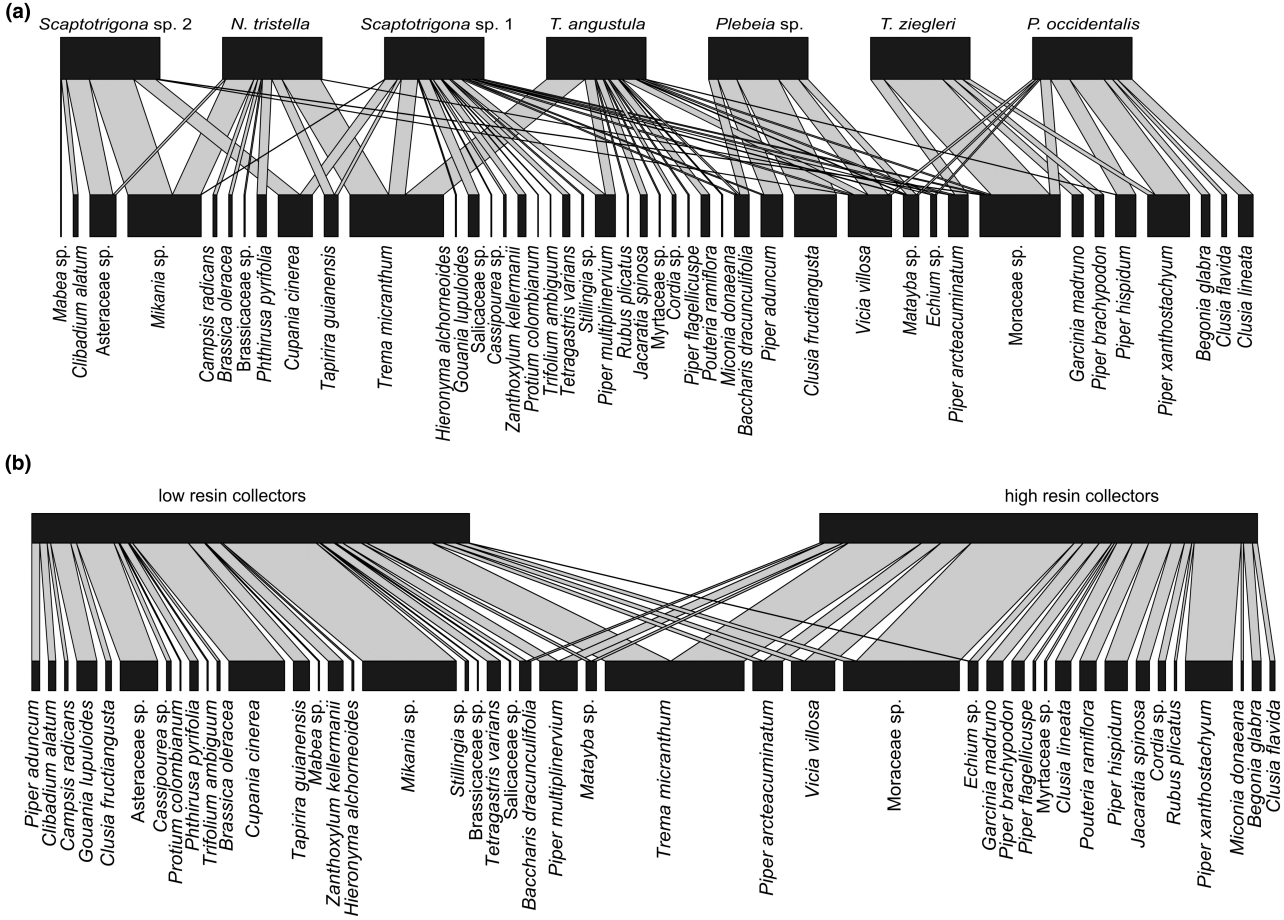
Pollen collection networks. Bipartite network for seven stingless bee species (top) and proportion of plant species (bottom) visited for pollen collection (a). Bipartite network showing stingless bee species pooled according to their resin collection preference (top) and proportion of plant species (bottom) visited for pollen collection (b). The bottom block width represents the overall proportions of a given plant species across stingless bee species pollen loads (Plant taxa accounting for more than 10% of reads are shown). *Nannotrigona tristella* is represented with 12 pollen samples, *Plebeia* sp. with 3, *P. occidentalis* with 9, *Scaptotrigona* sp. 1 with 29, *Scaptotrigona* sp. 2 with 20, *T. ziegleri* with 9, and *T. angustula* with 18.

Different stingless bee species collected pollen from different spectra of plant species (*H*_2_′ = 0.54, null‐model comparison: *p* < .001, Figure 6a) with some overlap. *Tetragona ziegleri* was the most specialized species (*d*′ = 0.75), followed by *Plebeia* sp. (*d*′ = 0.62), *Scaptotrigona* sp. 2 (*d*′ = 0.61), *P. occidentalis* (*d*′ = 0.59), *N. tristella* (*d*′ = 0.48), *T. angustula* (*d*′ = 0.39), and *Scaptotrigona* sp. 1 (*d*′ = 0.38).

From the taxa accounting for more than 10% of reads per sample, the three most visited plant families per species were for (a) *Nannotrigona tristella* (12 pollen samples): Cannabaceae (40% of all visited plants), Asteraceae (32%), Anacardiaceae (10%). (b) *Plebeia* sp. (3 samples): Clusiaceae (42%), Fabaceae (20%), Piperaceae (19%). (c) *P. occidentalis* (9 samples): Piperaceae (54%), Clusiaceae (20%), Moraceae (10%). (d) *Scaptotrigona* sp. 1 (29 samples): Cannabaceae (17%), Piperaceae (18%), Sapindaceae (15%). (e) *Scaptotrigona* sp. 2 (20 samples): Asteraceae (73%), Sapindaceae (22%), Moraceae (3%). (f) T. *ziegleri* (9 samples): Moraceae (59%), Piperaceae (30%), Clusiaceae (11%) and (g) *T. angustula* (18 samples): Cannabaceae (39%), Fabaceae (14%), and Piperaceae (13%).

**FIGURE 7:**
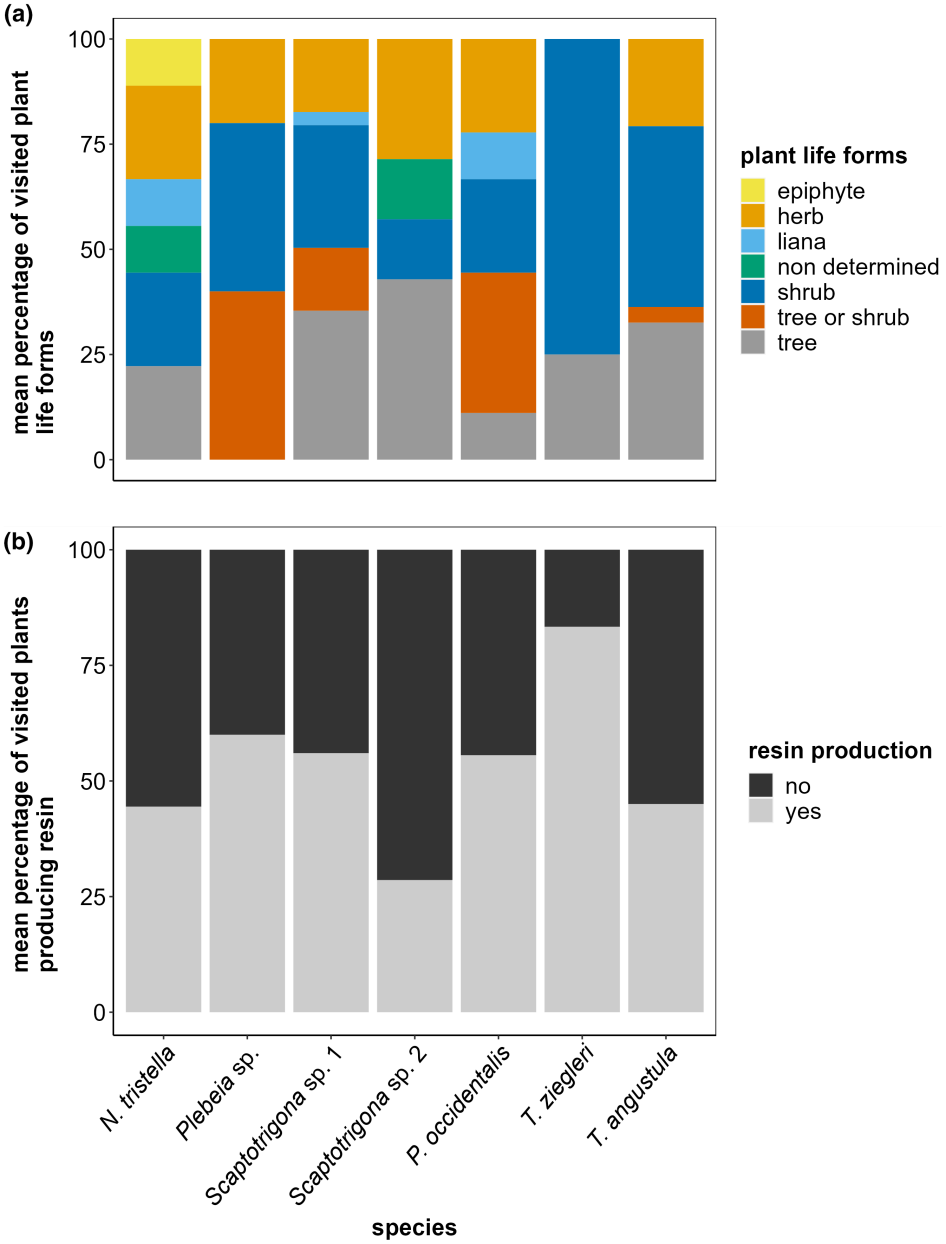
Proportions of plant life forms (a) and resin‐producing plants (b) (both mean percentages) within the spectrum of plants visited for pollen collection by the seven different stingless bee species observed in the forest of the reserves. Data comprise 12 pollen loads from *N. tristella* (low resin collector ‐lrc‐), 3 from Plebeia sp. (lrc), 9 from *P. occidentalis* (high resin collector ‐hrc‐), 29 from *Scaptotrigona* sp. 1 (lrc), 20 from *Scaptotrigona* sp. 2 (lrc), 9 from *T. ziegleri* (hrc), and 18 *T. angustula* (hrc).

Species did not differ in their preferences for herbs, shrubs, or trees (glm: likelihood‐ratio (LR) = 3.2, *p* = .79; LR = 5.9, *p* = .43; LR = 6.7, *p* = .3, respectively; Figure 7a). Across species, most of the pollen was collected from shrubs (14–40%), trees (11–40%), and herbs (20–30%), except for *T. ziegleri*, which collected pollen only from shrubs (75%) and trees (25%) (Figure 7a).

**FIGURE 8:**
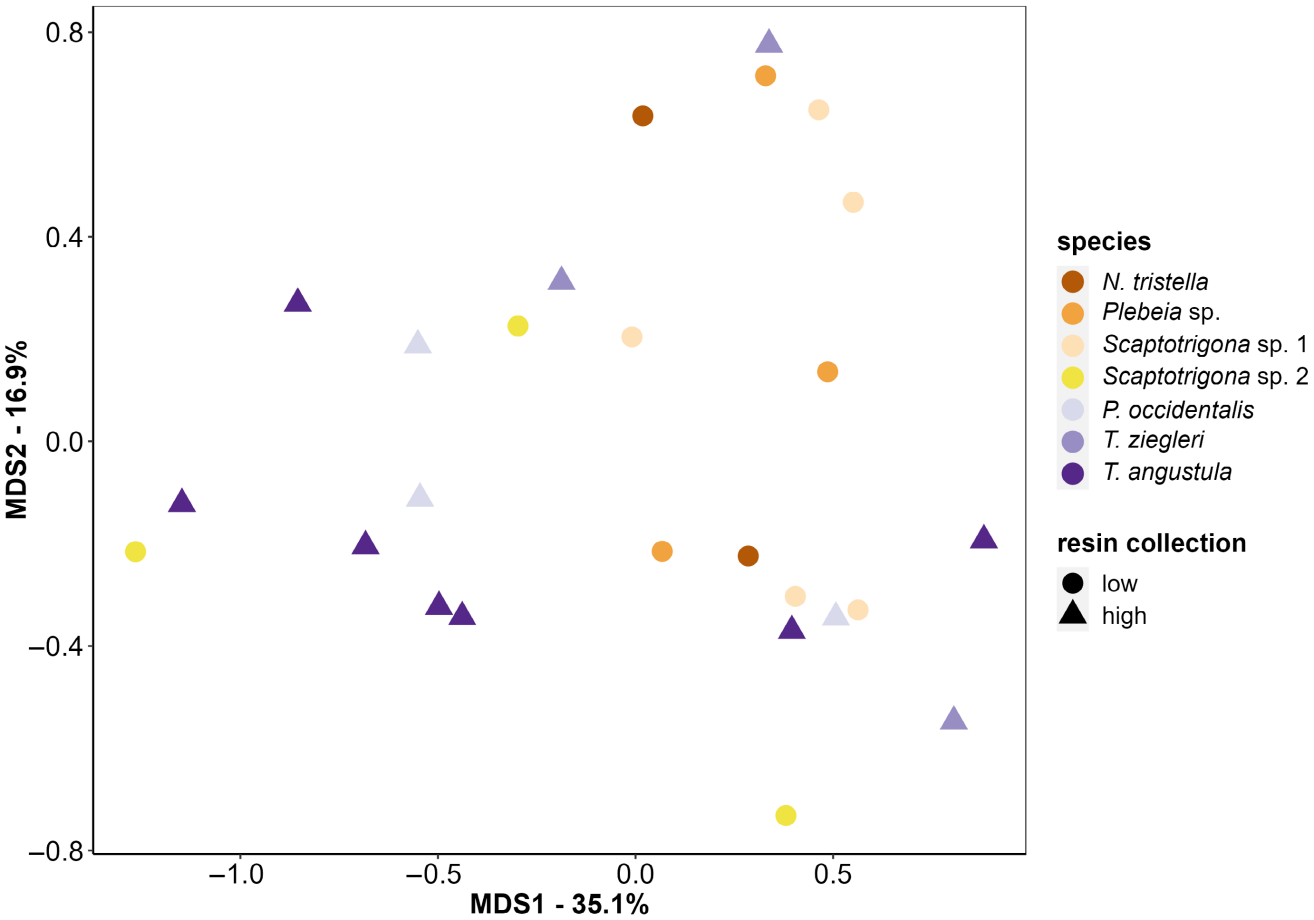
Multidimensional scaling based on Random Forest proximities, showing similarities in pollen amino acid profiles collected by the seven different stingless bee species in this study. Species are grouped into low (circles) and high (triangles) resin collectors. Different colors indicate different stingless bee species, and each dot represents one pooled pollen sample per colony and day. Except for *N. tristella* with *n* = 2, *Scaptotrigona* sp. 1 with *n* = 5 (2–3 from each colony), and *T. angustula* with *n* = 7 (2–3 from each colony), all other species with *n* = 3.

All bee species collected pollen with similar amino acid profiles (Figure 8, Figure S4; random forest out of the box (OOB) error rate estimate: 76.92%; class errors: 1.0 except for *Scaptotrigona* sp. 1 with a class error of 0.8 and *T. angustula* with 0.3). The amino acids with the highest percentages in at least one sample across pollen samples (i.e., equal or more than 10%) were leucine (range 8–50%), serine (7–15%), aspartic acid (0–28%), glycine (7–20%), and glutamic acid (0–12%). The total concentration of amino acids in pollen collected did not differ significantly between species (lm: *F*_6,19_ = 1.3, *p* = .31, Figure 5c).

### 3.3 Interaction between food and resin foraging

#### 3.3.1 Relationship between resin collection and general foraging patterns, resource intake, and nectar collection

High resin collectors tended to be two times more active (higher flight activity) than low resin collectors (lmm: *F*_1,2.8_ = 13.6, *p* = .04, Figure 3). Moreover, high resin collectors invested 15 and 180 times more into collecting only resin and resin and nectar, respectively, than low resin collectors (Table 3, Figure S3). Also, the high resin collectors tended to invest about four times more workers (per minute) into coming back with some resources, as well as three and two times fewer workers in collecting only nectar and in overall pollen collection, respectively (Table 3, Figure S3). The number of resin foragers/minute did not correlate with the number of pollen or nectar foragers/minute for any species (Table S6). The amount of sucrose collected, and sucrose intake/minute were similar for high and low resin collectors (glmmTMB: *χ* ^2^ = 0.007, *p* = .93; *χ* ^2^ = 0.18, *p* = .7, respectively, Figure 5a,b).

#### 3.3.2 Relationship between resin collection and visited plant species and pollen amino acid profiles

High and low resin collectors slightly differed in the spectrum of plant species visited for pollen collection (*H*_2_′ = 0.46, null‐model comparison: *p* < .001; *d*′ high resin collectors = 0.48; *d*′ low resin collectors = 0.44, Figure 6b). They did not differ in their preferences for pollen from herbs, shrubs, trees, or plants that additionally produce resin (glmm: *χ* ^2^ = 0.1, *p* = .78; *χ* ^2^ = 1.8, *p* = .19; *χ* ^2^ = 0.03, *p* = .85, *χ* ^2^ = 0.2, *p* = .70, respectively; Figure 7a,b).

The total concentration of amino acids in pollen was also similar (lmm: *F*_1,4.9_ = 1.5, *p* = .28, Figure 5c). However, pollen amino acid profiles largely, but not fully overlapped between the two groups (OOB error rate estimate: 42.3%; low resin collectors class error: 0.46; high resin collectors class error: 0.39; Figure 8, Figure S4). The amino acids mainly responsible for the partial separation of the two groups were glutamic acid, proline, alanine, isoleucine, and histidine (Figure S5, Table S7).

## 4 DISCUSSION

We examined variation in foraging patterns and resource nutritional composition of seven stingless bee species (11 wild colonies) in north‐western Ecuador based on field observations, DNA metabarcoding, and chemical analysis of pollen samples. As expected, the number of returning foragers per minute varied among species, as also observed by Leonhardt et al. (2007). Moreover, the species classified as high resin collectors based on their resin intake and the presence of resin compounds in their cuticular profiles tended to have more flights (per minute) than low resin collectors. This increase in activity could be a strategy to compensate for their comparatively lower proportion of food foragers, as they might have to trade‐off collecting resin and collecting food resources. Note that we occasionally observed foragers returning with both pollen and resin, which were most likely pollen foragers with minute resin residues from previous foraging trips or as part of their surface profile (Leonhardt et al., 2009), as pollen mixed with resin is unusable as food (Armbruster, 1984). In fact, the observed increase in activity rendered the workforce invested only in pollen collection similar across all species and between high and low resin collectors. Consequently, high resin collectors also tended to invest more workforce into collecting resources overall (note that the surface of the bees' hindlegs often appears sticky due to their unique surface profile and can be mistaken for a resin load, which might have led to slightly underestimating the number of foragers with actually no load).

The percentage of nectar foragers and sucrose amount in nectar also differed between some species, which is contrary to observations on Sumatran and Australian stingless bees (Inoue et al., 1985; Leonhardt et al., 2014). Our study species and colonies also differed in collected sucrose amounts, with the *Ptilotrigona occidentalis* colony collecting nectar with the highest sucrose content. Sucrose amounts observed ranged from 0.12 to 2.4 mg (percentage 14–72% sucrose), which is similar to reports for other stingless bee species (Inoue et al., 1985; Leonhardt et al., 2007; Roubik & Buchmann, 1984). We additionally observed differences between species in their sucrose intake per minute, with the *Ptilotrigona occidentalis* colony also showing the highest sucrose intake. Differences in sucrose intake have also been reported among Australian stingless bees (Leonhardt et al., 2014). Contrary to our expectations, sucrose intake was not higher in stingless bees with a comparatively higher foraging activity as observed for *Tetragonula carbonaria* in Australia (Leonhardt et al., 2014).

The observed differences in sucrose collection might be related to body size and/or honey storage. For example, *P. occidentalis* collected double the amount of sucrose per minute than the other species. This species has a comparatively large body size with a head width of 3 mm (Lichtenberg et al., 2017) and may therefore require more sucrose than smaller species. The genus *Ptilotrigona* stores generally less honey than other species (Camargo & Pedro, 2004) and might thus need to compensate for this by collecting higher amounts of sucrose. However, low honey storage has also been reported for the genus *Nannotrigona* (Rasmussen & Gonzalez, 2017), but our *N. tristella* colony did not show a higher sucrose intake compared to the other colonies, suggesting that additional factors might explain the observed difference in sucrose intake.

Also contrary to our initial expectations, we did not find significant differences in the amount nor sucrose intake per minute between high and low resin collectors. This suggests that an increased resin intake might not necessarily influence carbohydrate intake. Note that we included all returning foragers that came back with nectar in these analyses. However, stingless bees often leave their nest with some nectar in their crops (typically less than 1 μL, Inoue et al., 1985; Leonhardt et al., 2007). As we did not check for amounts of nectar in departing foragers, we cannot entirely rule out having included a few foragers returning without an actual load and thus slightly overestimating the number of nectar foragers.

As previously reported and expected, we found that our studied stingless bee species collected pollen from various plant species, but that each species collected most of its pollen from only three plant families as already reported by Vossler (2018) and Garcia Bulle Bueno et al. (2023). Piperaceae, Clusiaceae, Moraceae, and Cannabaceae were the most commonly visited families. Also, some species (i.e., *T. ziegleri*, *Plebeia* sp., and *Scaptotrigona* sp. 2) collected pollen from fewer plants, indicating that they might prefer pollen from particular plants. Contrary to our expectations, the differences between the visited plants were not driven by the plants' life form nor by pollen protein content and amino acid profile. Instead, different species mostly overlapped in their pollen amino acid profiles which has also been observed in bumblebees (Kriesell et al., 2017), suggesting that they share similar requirements. However, we often had only few pollen samples per species and a limited number of colonies per species. Moreover, we did not check for the foraging range of our studied species or the plant species which were flowering at the time of the study. We therefore refrain from drawing any conclusions on species‐specific foraging choices or preferences.

Interestingly, high and low resin collectors foraged pollen from slightly different spectra of plants, which was independent of the plants' life forms or whether the plants additionally produced resin, suggesting that the bees do not preferentially collect pollen from plants that additionally produce resin. Moreover, amino acid profiles of pollen collected by the two groups tended to differ, indicating that a high resin intake may affect the spectrum of plants visited for pollen foraging. However, the high resin collectors also show a solitary foraging strategy, while, for example, species of the genus *Scaptotrigona* show forager recruitment (Table 1). Some *Nannotrigona* and *Plebeia* species are considered solitary foragers (Aguilar et al., 2005; Lichtenberg et al., 2010; Slaa et al., 1997). However, they have also been observed recruiting nestmates to food sources, and *Plebeia tica* appears to be a more effective recruiter than *T. angustula* (Aguilar et al., 2005). These differences in recruitment strategies may also explain the differences in visited plant and pollen amino acid profiles as observed in our study. Group foragers and mass recruiter species tend to dominate food patches, often driving off solitary foragers, which then visit other plants or access resources before the dominant species arrive (Cairns et al., 2005; Nagamitsu & Inoue, 1997). Future research should ideally compare recruiting species differing in resin intake to disentangle effects of foraging strategies and resin.

It is noteworthy that species classified here as high resin collectors (*P. occidentalis* and *T. angustula*) rely more heavily on resin for defense (see Table 1) compared to low resin collectors. For instance, the high resin collector *T. angustula* applies resin to predators and places resin droplets around the nest entry to trap intruders (Wittmann, 1985, C. Rasmussen *pers. obs*.). In contrast, the low resin collector *N. tristella* tends to hide in it is nest and seals the nest entry with wax (Rasmussen & Gonzalez, 2017, G. N. Villagómez *pers. obs*.). Species within the *Scaptotrigona* genus exhibit strong physical aggression when threatened, such as attacking and biting intruders (e.g., Couvillon et al., 2008; Jungnickel et al., 2004; G. N. Villagómez *pers. obs*.). These differences in defense strategies may partially explain the significant effort invested in resin collection by the high resin collectors.

Additional limitations of our study are the limited number of observed colonies per species and that all observations were conducted in a single season. Environmental and colony‐specific factors, such as predator pressure, a colony's developmental or feeding stage, time of day, and season, affect resource collection and resource intake by colonies, including resin collection (Hilário et al., 2000, 2012; Hofstede & Sommeijer, 2006; Leonhardt & Blüthgen, 2009; Newis et al., 2023; Nunes‐Silva et al., 2010; Shanahan & Spivak, 2021). To fully elucidate the effect of resin collection on stingless bee foraging and resource intake, repeated observations across different times of the year and including additional colonies as well as controlled experiments that manipulate resin and pollen storage within nests (e.g., Newis et al., 2023) are needed.

To conclude, our findings highlight the importance of studying the foraging ecology and resource intake of different stingless bee species in their natural environment to understand how differences in life‐history traits may affect species' foraging patterns. Such differences may be innate or a consequence of specific colony states (e.g., colony reserves, developmental stage) (Biesmeijer & Slaa, 2004). As we have only a few colonies for some species, we cannot disentangle these two factors. We do show, however, that the collection of a non‐food resource, such as resin, appears to affect stingless bee foraging patterns, plant choices, and resource intake. Plant resins are an ideal material for construction and defense (Armbruster, 1984; Chui et al., 2022; Greco et al., 2010; Roubik, 2006), and can even be incorporated into their surface profile as protection against predation and microbes (Leonhardt, 2017). They have therefore become a vital resource for many stingless bee species. Their importance could have led to species modifying their foraging behavior, that is, increasing their overall foraging activity to secure sufficient resin intake without putting food intake at risk. The often‐neglected importance of resin clearly calls for more research focusing on resin collection and use by stingless bees, for example, of colonies located in altered forests where probably fewer or different resin resources are available.

## AUTHOR CONTRIBUTIONS

**Gemma Nydia Villagómez:** Conceptualization (supporting); data curation (lead); formal analysis (lead); investigation (equal); writing – original draft (lead). **Alexander Keller:** Conceptualization (equal); formal analysis (equal); funding acquisition (equal); methodology (equal); resources (equal); supervision (equal); writing – review and editing (equal). **Claus Rasmussen:** Formal analysis (equal); methodology (equal); supervision (supporting); writing – review and editing (equal). **Pablo Lozano:** Formal analysis (supporting); writing – review and editing (equal). **David A. Donoso:** Validation (equal); writing – review and editing (equal). **Nico Blüthgen:** Methodology (supporting); supervision (equal); validation (equal); writing – review and editing (equal). **Sara Diana Leonhardt:** Conceptualization (lead); formal analysis (supporting); methodology (supporting); project administration (equal); resources (equal); supervision (lead); validation (equal); writing – review and editing (lead).

## CONFLICT OF INTEREST STATEMENT

The authors reported no conflict of interest.

## Supporting information


Appendix S1.

Data S1.

## Data Availability

The data that support the findings of this study are openly available in the online repository OSF, DOI: 10.17605/OSF.IO/N836S. Pollen metabarcoding data can be found in NCBI BioProject ID: PRJNA1019327. https://www.ncbi.nlm.nih.gov/bioproject/PRJNA1019327.
