# Supplementary material for: Nutrients or resin? – The relationship between resin and food foraging in stingless bees
Source: Ecol Evol. 2024 Feb 8;14(2):e10879. doi: 10.1002/ece3.10879 (PMC10853648; doi:10.1002/ece3.10879)
Supplement: Supplementary file 1 — Appendix S1. [file ECE3-14-e10879-s001.docx]

**Supplementary information**

**Food or defense? – The effect of resin collection on stingless bee foraging**

Gemma Nydia Villagómez^1^, Alexander Keller^2^, Claus Rasmussen^3^, Pablo Lozano^4^, David A. Donoso^5,6^, Nico Blüthgen^7^, Sara Diana Leonhardt^8^

^1^ University of Würzburg, Biocenter, Department of Animal Ecology and Tropical Biology, Am Hubland, 97074 Würzburg, Germany.

^2^ LMU Munich, Faculty of Biology, Cellular and Organismic Networks, 82152 Planegg-Martinsried, Germany

^3^Aarhus University, Department of Agroecology, 8830 Tjele, Denmark.

^4^ Universidad Estatal Amazónica, 160-150 Puyo, Ecuador

^5^ Escuela Politécnica Nacional, Departamento de Biología, E11 253 Quito, Ecuador

^6^ Universidad Tecnológica Indoamérica, Centro de Investigación de la Biodiversidad y Cambio Climático, EC170103 Quito, Ecuador

^7^Technical University of Darmstadt, Department of Biology, Ecological Networks, Schnittspahnstr. 3, 64287 Darmstadt, Germany

^8^Technical University of Munich, TUM School of Life Sciences, Plant-Insect-Interactions, 85354 Freising, Germany.

Corresponding author: [sara.leonhardt@tum.de](mailto:sara.leonhardt@tum.de)

# Analytical details

## Stingless bee DNA barcoding and pollen metabarcoding

Stingless bee DNA extraction and barcoding were carried out by Advanced Identification Methods GmbH (Leipzig, Germany) using Sanger sequencing of the COI gene with LCO and HCO primers. Identification was performed by matching the obtained sequence with sequences in the BOLD Identification System (Ratnasingham & Hebert, 2007).

The DNA from the pollen was extracted using the protocol from Machery-Nagel (Düren, Germany) NucleoSpin Food Kit. Pollen DNA metabarcoding of the ITS2 plant region was performed in house following Sickel et al., (2015) with the amplifying primers ITS-S2F and ITS4R to include dual indices and Ilumina Adapters. We used 17 forward index sequences SA501–SB508, SC501 and 24 reverse indices SA701–SB712. Each sample was assigned a different forward/reverse index combination for specific labelling.

PCR amplification conditions: initial denaturation at 95°C for 4 min, 37 cycles of denaturation at 95°C for 40 s, annealing at 49°C for 40 s and extension at 72°C for 40 s; followed by a final extension step at 72°C for 5 min (Sickel et al., 2015).

Thirty-three pollen samples could not be amplified with the previous amplification conditions, so they were amplified using different PCR conditions: initial denaturation at 96°C for 3 min, 37 cycles of denaturation at 96°C for 10 s, annealing at 49°C for 40 s and extension at 72°C for 40 s; followed by a final extension step at 72°C for 7 min.

To ensure similar DNA amounts for sequencing, DNA amounts in each PCR product were normalized using the SequalPrep™ Normalization Plate Kit (Invitrogen GmbH, Darmstadt, Germany). Quality control and quantification were performed using a Bioanalyzer High Sensitivity DNA Chip (Agilent Technologies, Santa Clara, CA, USA) and dsDNA High Sensitivity Assay (Life Technologies GmbH, Darmstadt, Germany). Sequencing was performed on the Illumina MiSeq using 2 x 250 cycles v2 chemistry (Illumina Inc., San Diego, CA, USA).

We used VSEARCH v2.14.2 (Rognes, Flouri, Nichols, Quince, & Mahé, 2016) to join paired ends of forward and reverse reads. We also used VSEARCH to remove reads shorter than 150bp, quality filtering (EE < 1, (Edgar & Flyvbjerg, 2015)), de-novo chimera filtering (following UCHIME3; Edgar, 2016b), and determination of amplicon sequence variants (ASVs) (Edgar, 2016b), as previously done for pollen metabarcoding networks (Elliott et al., 2021). Reads were first directly mapped with global alignments using VSEARCH against a floral ITS2 reference database for the study region and an identity cut-off threshold of 97%. This database was created with the BCdatabaser (Keller et al., 2020) and with a list of potential plants that could be present in the study region (Supplementary information “Potential plants in the Chocó region”). For still unclassified reads, we used SINTAX (Edgar, 2016a) to assign taxonomic levels as deep as possible with a global reference database (Keller et al., 2015; Ankenbrand, Keller, Wolf, Schultz, & Förster, 2015).

## Analysis of pollen amino acids

The method consists of mixing each sample with 100 μL water, placing it for 30 min. in an ultrasonic bath, then in the fridge for 1 hour before centrifuging it at 11,400 rpm for 7 min. Afterwards, the sediment was dissolved in 200 μL 6 N HCl and heated up to 100 °C for 4 hrs. The samples were cooled down at room temperature and centrifuged at 18,000 rpm for 10 min. The supernatants were then transferred to a new tube, evaporated at 100 °C, redissolved in 200 μL water, and centrifuged again for 10 min. One hundred μL of the supernatant were mixed with 20 μL of 12.5 % sulfosalicylic acid and extracted in the refrigerator for 30 min. After short mixing and centrifuging for another 10 min., 100 μL of the supernatant were mixed with 100 μL of sample rarefaction buffer in a new tube, centrifuged and membrane filtered at 10,000 rpm, and finally stored for further analysis by IEC.

## Cuticular profile extraction

Five foragers of each nest were captured and frozen. Afterward each of them was placed in a gas chromatography (GC) vial with hexane for two minutes, before taking out the forager. The hexane extract was then frozen and stored for its later chemical analysis. The extracts were analysed via gas chromatography/mass spectronomy (GC: 7890A, MSD: 5975 inert XL, Agilent Technologies, Santa Clara, USA) using helium as carrier gas. One µl of each sample was injected in splitless mode at 300 °C. The initial oven temperature was set at 60 °C. After 1 min, temperature was increased to 300°C at 5°C/minute and held for 10 minutes. Electron ionization mass spectra were recorded from 40 to 600 m/z. MS Source was 230 °C. For data acquisition, we used the software MSD ChemStation F.01.00.1903 (Agilent Technologies, Böblingen, Germany). Cuticular compounds were classified in four groups (sesquiterpenoids, diterpenoids, triterpenoids and cuticular hydrocarbons) according to their retention times and by comparing their peaks to respective compounds in the library NIST MS Search 2.0. Terpenoids are derived from resin (see Drescher et al., 2019) and typical for high resin collectors, while cuticular hydrocarbons (CHCs) comprise compounds (e.g. alkanes, alkenes, esters etc.) produced by the bees themselves.

# Statistical analysis

### **Bee species-specific differences**

#### **Bee species-specific differences in foraging patterns, resource intake, and nectar collection**

Using Akaike’s information criterion (AIC) for model selection, we assessed if colony ID and (or) nest location (i.e., reserve) explained a proportion of the variation in our response variables and should therefore be included as random factors in the models. The AICs of the models presented in this section in the manuscript (except for the glms with quasibinomial distribution which does not provide AIC) were compared with the AIC of: (a) a model including colony ID and reserve as random factors, (b) a model including only reserve as a random factor, and (c) a model including only colony ID as a random factor. In all cases, models without random factor(s) had smaller AIC values (Table S 2), indicating that adding random factors did not improve the explanatory force of our models and that intraspecific differences and nest location did not crucially affect model results and could be excluded (Figure S 2 shows an example of similar percentage of foragers for each resource for different colonies of the same species).

#### **Bee species-specific differences in plant species visited and in pollen amino acid profiles**

We used bee-pollen-based interaction networks to depict bee species-specific preferences and specialization levels on pollen sources. In this analysis we calculated the quantitative network-level specialization index, H2', and the species-level specialization index, d’ (Blüthgen, Menzel, & Blüthgen, 2006) (bipartite R-package; Dormann et al., 2008). To obtain d’ values relative data were multiplied by 100,000,000 and rounded to obtain integers. Using these integers values we used the null-model approach (number of random models = 1000, and the method "r2dtable", which generates random networks keeping row and column sums constant) to see if our obtained H2' indeces were significantly different from random networks. *P*-values were obtained by counting the proportion of the H2’ from null-modeled H2’ that exceeded the value of the observed H2’ following Dormann, (2022).

### **Effect of resin collection**

#### **Effect of resin collection on visited plant species and pollen amino acid profiles**

To test for compositional differences between high and low resin collectors in proportional pollen amino acid profiles, we conducted an additional classification analysis based on Breiman's random forest algorithm (function randomForest, 100,000 trees) using resin collection as the classification predictor. We obtained the out-of-bag (OOB) estimate of error rate and the class errors for each group. Additionally, to test which amino acids were more important for the classification, we calculated Mean Decrease Accuracy Indexes for all of them. This index indicates how much the model accuracy decreases when a specific amino acid is dropped, the higher the value the more important was this amino acid to differentiate between high and low resin collectors.

# Tables

## Plant’s life forms and resin production

**Table S 1**. List of the life forms (i.e., epiphyte, tree, shrub, herb, liana, tree or shrub), and resin production of the taxa accounting for more than 10% of reads per sample. The reference from where it was seen if the plants produce resin is given.

| Order | Family | Genus | species | Life form | Resin production | Resin production reference |
| --- | --- | --- | --- | --- | --- | --- |
| Asterales | Asteraceae | Asteraceae sp. | Asteraceae sp. | indeterminate | 1 | (Villagra, Meza, & Urzúa, 2014) |
| Asterales | Asteraceae | *Baccharis* | *Baccharis dracunculifolia* | shrub | 1 | (Fernandes et al., 2018) |
| Cucurbitales | Begoniaceae | *Begonia* | *Begonia glabra* | liana | 0 |  |
| Brassicales | Brassicaceae | *Brassica* | *Brassica oleracea* | herb | 0 |  |
| Brassicales | Brassicaceae | Brassicaceae sp. | Brassicaceae sp. | herb | 0 |  |
| Lamiales | Bignoniaceae | *Campsis* | *Campsis radicans* | liana | 0 |  |
| Malpighiales | Rhizophoraceae | *Cassipourea* | *Cassipourea* sp. | tree | 1 | (Burkill, 1985) |
| Asterales | Asteraceae | *Clibadium* | *Clibadium alatum* | herb | 0 |  |
| Malpighiales | Clusiaceae | *Clusia* | *Clusia flavida* | tree or shrub | 1 | (Alencar, Tölke, & Mayer, 2020) |
| Malpighiales | Clusiaceae | *Clusia* | *Clusia fructiangusta* | tree or shrub | 1 | (Alencar et al., 2020) |
| Malpighiales | Clusiaceae | *Clusia* | *Clusia lineata* | tree or shrub | 1 | (Alencar et al., 2020) |
| Boraginales | Cordiaceae | *Cordia* | *Cordia* sp. | shrub | 1 | (Paniagua-Zambrana, Bussmann, & Romero, 2020) |
| Sapindales | Sapindaceae | *Cupania* | *Cupania cinerea* | tree | 0 |  |
| Boraginales | Boraginaceae | *Echium* | *Echium* sp. | herb | 1 | (Carlquist, 1970) |
| Malpighiales | Clusiaceae | *Garcinia* | *Garcinia madruno* | tree | 1 | (Hammel, 1989; Alencar et al., 2020) |
| Rosales | Rhamnaceae | *Gouania* | *Gouania lupuloides* | liana | 0 |  |
| Malpighiales | Phyllanthaceae | *Hieronyma* | *Hieronyma alchorneoides* | tree | 1 | (Vignote Peña, 2014) |
| Brassicales | Caricaceae | *Jacaratia* | *Jacaratia spinosa* | tree | 0 |  |

**Table S1 (cont.)**

| Order | Family | Genus | species | Life form | Resin production | Resin production reference |
| --- | --- | --- | --- | --- | --- | --- |
| Malpighiales | Euphorbiaceae | *Mabea* | *Mabea* sp. | tree | 0 |  |
| Sapindales | Sapindaceae | *Matayba* | *Matayba* sp. | tree or shrub | 0 |  |
| Myrtales | Melastomataceae | *Miconia* | *Miconia donaeana* | shrub | 0 |  |
| Asterales | Asteraceae | *Mikania* | *Mikania* sp. | shrub | 1 | (Amorin, de Paula, da Silva, Farago, & Budel, 2014) |
| Rosales | Moraceae | Moraceae sp. | Moraceae sp. | tree | 0 |  |
| Myrtales | Myrtaceae | Myrtaceae sp. | Myrtaceae sp. | tree | 1 | (Padovan, Keszei, Külheim, & Foley, 2014) |
| Santalales | Loranthaceae | *Phthirusa* | *Phthirusa pyrifolia* | epiphyte | 0 |  |
| Piperales | Piperaceae | *Piper* | *Piper aduncum* | shrub | 1 | (Hirschhorn, 1981) |
| Piperales | Piperaceae | *Piper* | *Piper arcteacuminatum* | shrub | 1 | (Hirschhorn, 1981) |
| Piperales | Piperaceae | *Piper* | *Piper brachypodon* | shrub | 1 | (Hirschhorn, 1981) |
| Piperales | Piperaceae | *Piper* | *Piper flagellicuspe* | shrub | 1 | (Hirschhorn, 1981) |
| Piperales | Piperaceae | *Piper* | *Piper hispidum* | shrub | 1 | (Hirschhorn, 1981) |
| Piperales | Piperaceae | *Piper* | *Piper multiplinervium* | shrub | 1 | (Hirschhorn, 1981) |
| Piperales | Piperaceae | *Piper* | *Piper xanthostachyum* | shrub | 1 | (Hirschhorn, 1981) |
| Ericales | Sapotaceae | *Pouteria* | *Pouteria ramiflora* | tree | 0 |  |
| Sapindales | Burseraceae | *Protium* | *Protium colombianum* | tree | 1 | (Deharo et al., 2001; Araujo-Murakami & Zenteno Ruiz, 2006) |
| Rosales | Rosaceae | *Rubus* | *Rubus plicatus* | shrub | 0 |  |
| Malpighiales | Salicaceae | Salicaceae sp. | Salicaceae sp. | tree or shrub | 1 | (Curtis & Lersten, 1974; Thadeo, Azevedo, & Meira, 2014) |
| Malpighiales | Euphorbiaceae | *Stillingia* | *Stillingia* sp. | shrub | 0 |  |

**Table S1 (cont.)**

| Order | Family | Genus | species | Life form | Resin production | Resin production reference |
| --- | --- | --- | --- | --- | --- | --- |
| Sapindales | Anacardiaceae | *Tapirira* | *Tapirira guianensis* | tree | 1 | (Dussourd & Denno, 1991) |
| Sapindales | Burseraceae | *Tetragastris* | *Tetragastris varians* | tree | 1 | (Zoghbi, Andrade, Santos, Luz, & Maia, 1998) |
| Rosales | Cannabaceae | *Trema* | *Trema micranthum* | tree | 0 |  |
| Fabales | Fabaceae | *Trifolium* | *Trifolium ambiguum* | herb | 0 |  |
| Fabales | Fabaceae | *Vicia* | *Vicia villosa* | herb | 0 |  |
| Sapindales | Rutaceae | *Zanthoxylum* | *Zanthoxylum kellermanii* | tree or shrub | 1 | (Ferreira et al., 2011) |

## Statistical results

**Table S 2.** Akaike’s information criterion (AIC) values from the models that tested differences between stingless bee species on their returning foragers per minute with each of the studied resources and on their sucrose intake (i.e., sucrose amount collected, and sucrose intake per minute). The models that were compared were: (a) models including colony ID and reserve as random factors (“all”), (b) models including only reserve (i.e., location) as a random factor (“reserve”), (c) models including only colony ID as a random factor (“colony”), and (d) models without random factor(s) (“without”). Note that the models without random factor(s) are the models with the smaller AIC values.

|  | Models’ AIC | | | |
| --- | --- | --- | --- | --- |
|  | **without** | **colony** | **reserve** | **all** |
| Models of returning foragers per minute with: |  |  |  |  |
| no load | -54.94 | -52.94 | -52.94 | -50.94 |
| only nectar | -23.24 | -21.24 | -21.24 | -19.24 |
| only pollen | -54.86 | -53.85 | -52.57 | -51.85 |
| only resin | -44.79 | -42.79 | -42.79 | -40.79 |
| pollen and nectar | -24.80 | -22.80 | -22.81 | -20.80 |
| resin and nectar | -21.77 | -19.77 | -19.77 | -17.77 |
| total pollen | -44.29 | -42.29 | -42.29 | -40.29 |
| Sucrose intake |  |  |  |  |
| sucrose amount | 119.23 | 121.23 | 121.28 | 123.23 |
| sucrose intake per minute | -533.11 | -531.11 | -531.11 | -529.11 |

**Table S 3**. Pairwise comparisons between stingless bee species in the foraging activity (norm. foragers/min.). Shown are the estimate, t-ratios and p-values obtained with contrasts of estimated marginal means (adjustment method: Holm; emmeans R package). Bold values indicate significant differences between species (p ≤ 0.05).

|  | Activity (foragers/minute) | | |
| --- | --- | --- | --- |
| contrast | *estimate* | *t-ratio* | *p* |
| *N. tristella* / *Plebeia* sp. | -0.28 | -2.26 | 0.38 |
| *N. tristella* / *P. occidentalis* | -0.35 | -2.81 | 0.13 |
| *N. tristella* / *Scaptotrigona* sp. 1 | -0.01 | -0.08 | 1.00 |
| *N. tristella* / *Scaptotrigona* sp. 2 | -0.07 | -0.55 | 1.00 |
| *N. tristella* / *T. ziegleri* | -0.28 | -2.65 | 0.19 |
| *N. tristella* / *T. angustula* | -0.33 | -3.30 | **0.04** |
| *Plebeia* sp. / *P. occidentalis* | -0.07 | -0.55 | 1.00 |
| *Plebeia* sp. / *Scaptotrigona* sp. 1 | 0.27 | 2.53 | 0.22 |
| *Plebeia* sp. / *Scaptotrigona* sp. 2 | 0.21 | 1.71 | 0.95 |
| *Plebeia* sp. / *T. ziegleri* | 0.00 | -0.05 | 1.00 |
| *Plebeia* sp. / *T. angustula* | -0.05 | -0.53 | 1.00 |
| *P. occidentalis* / *Scaptotrigona* sp. 1 | 0.34 | 3.17 | 0.06 |
| *P. occidentalis* / *Scaptotrigona* sp. 2 | 0.28 | 2.27 | 0.38 |
| *P. occidentalis* / *T. ziegleri* | 0.06 | 0.60 | 1.00 |
| *P. occidentalis* / *T. angustula* | 0.02 | 0.15 | 1.00 |
| *Scaptotrigona* sp. 1 / *Scaptotrigona* sp. 2 | -0.06 | -0.55 | 1.00 |
| *Scaptotrigona* sp. 1 / *T. ziegleri* | -0.27 | -3.15 | 0.06 |
| *Scaptotrigona* sp. 1 / *T. angustula* | -0.32 | -4.06 | **0.01** |
| *Scaptotrigona* sp. 2 / *T. ziegleri* | -0.22 | -2.02 | 0.55 |
| *Scaptotrigona* sp. 2 / *T. angustula* | -0.26 | -2.63 | 0.19 |
| *T. ziegleri* / *T. angustula* | -0.05 | -0.61 | 1.00 |

**Table S 4.** Pairwise comparison between the stingless bee species in the proportion (prop.) and number of returning foragers per minute (calculated with the normalized activity) with each resource. Shown are z-, t- ratios and p-values obtained with contrasts of estimated marginal means (adjustment method: Holm; emmeans R package). Bold values indicate significant differences between species (p ≤ 0.05).

|  | no load | | | | only nectar | | | | only pollen | | | |
| --- | --- | --- | --- | --- | --- | --- | --- | --- | --- | --- | --- | --- |
|  | **prop.** | | **foragers/min.** | | **prop.** | | **foragers/min.** | | **prop.** | | **foragers/min.** | |
| contrast | *z* | *p* | *t* | *p* | *z* | *p* | *t* | *p* | *z* | *p* | *t* | *p* |
| *N. tristella* / *Plebeia* sp. | 0.1 | 1 | -0.7 | 1 | 0.03 | 1 | -1 | 1 | 0.5 | 1 | -0.6 | 1 |
| *N. tristella* / *P. occidentalis* | 3.6 | **0.005** | 1.9 | 0.74 | 1.8 | 0.87 | 0.7 | 1 | 1.1 | 1 | -1.3 | 1 |
| *N. tristella* / *Scaptotrigona* sp. 1 | 2.3 | 0.25 | 2.5 | 0.24 | 3.0 | **0.04** | 1.6 | 1 | -0.7 | 1 | -0.8 | 1 |
| *N. tristella* / *Scaptotrigona* sp. 2 | 2.6 | 0.12 | 2.3 | 0.34 | 1.2 | 1 | -0.2 | 1 | -1.9 | 0.82 | -2 | 0.98 |
| *N. tristella* / *T. ziegleri* | 4.8 | **<0.0001** | 2.6 | 0.19 | 3.8 | **0.003** | 3.0 | 0.08 | 1.8 | 0.90 | -0.05 | 1 |
| *N. tristella* / *T. angustula* | 5.2 | **<0.0001** | 3.5 | **0.02** | 3.8 | **0.003** | 2.1 | 0.53 | 0.8 | 1 | -1.0 | 1 |
| *Plebeia* sp. / *P. occidentalis* | 3.2 | **0.02** | 2.6 | 0.21 | 1.5 | 1 | 1.7 | 1 | 0.4 | 1 | -0.7 | 1 |
| *Plebeia* sp. / *Scaptotrigona* sp. 1 | 1.7 | 0.58 | 3.3 | **0.03** | 2.5 | 0.17 | 2.5 | 0.27 | -1.1 | 1 | -0.1 | 1 |
| *Plebeia* sp. / *Scaptotrigona* sp. 2 | 2.2 | 0.28 | 3 | 0.07 | 1 | 1 | 0.8 | 1 | -2.0 | 0.73 | -1.4 | 1 |
| *Plebeia* sp. / *T. ziegleri* | 4.2 | **0.001** | 3.3 | **0.03** | 3.5 | **0.01** | 3.8 | **0.01** | 0.8 | 1 | 0.6 | 1 |
| *Plebeia* sp. / *T. angustula* | 4.3 | **0.0003** | 4.4 | **0.001** | 3.2 | **0.03** | 3.1 | **0.06** | -0.1 | 1 | -0.3 | 1 |
| *P. occidentalis* / *Scaptotrigona* sp. 1 | -2.3 | 0.25 | -0.1 | 1 | 1 | 1 | 0.8 | 1 | -1.9 | 0.82 | 0.7 | 1 |
| *P. occidentalis* / *Scaptotrigona* sp. 2 | -1.5 | 0.86 | 0.1 | 1 | -0.5 | 1 | -0.9 | 1 | -3 | 0.06 | -0.6 | 1 |
| *P. occidentalis* / *T. ziegleri* | 0.5 | 1 | 0.5 | 1 | 2.5 | 0.20 | 2.3 | 0.37 | 0.5 | 1 | 1.3 | 1 |
| *P. occidentalis* / *T. angustula* | 0.01 | 1 | 0.8 | 1 | 1.7 | 0.97 | 1.3 | 1 | -0.6 | 1 | 0.6 | 1 |
| *Scaptotrigona* sp. 1 / *Scaptotrigona* sp. 2 | 0.9 | 1 | 0.2 | 1 | -1.6 | 0.97 | -1.8 | 1 | -1.6 | 1 | -1.5 | 1 |
| *Scaptotrigona* sp. 1 / *T. ziegleri* | 3.3 | **0.01** | 0.7 | 1 | 1.8 | 0.87 | 1.7 | 1 | 2.9 | 0.06 | 0.8 | 1 |
| *Scaptotrigona* sp. 1 / *T. angustula* | 3.4 | **0.01** | 1.3 | 1 | 0.6 | 1 | 0.4 | 1 | 1.9 | 0.82 | -0.2 | 1 |
| *Scaptotrigona* sp. 2 / *T. ziegleri* | 2.2 | 0.26 | 0.4 | 1 | 2.9 | 0.07 | 3.2 | **0.05** | 4 | **0.001** | 2.1 | 0.84 |
| *Scaptotrigona* sp. 2 / *T. angustula* | 2 | 0.41 | 0.9 | 1 | 2.3 | 0.28 | 2.4 | 0.34 | 3.3 | **0.02** | 1.4 | 1 |
| *T. ziegleri* / *T. angustula* | -0.6 | 1 | 0.3 | 1 | -1.4 | 1 | -1.5 | 1 | -1.4 | 1 | -1.1 | 1 |

***Table S 4.*** *(cont.)*

|  | only resin | | | | pollen and nectar | | | | resin and nectar | | | |
| --- | --- | --- | --- | --- | --- | --- | --- | --- | --- | --- | --- | --- |
|  | **prop.** | | **foragers/min.** | | **prop.** | | **foragers/min.** | | **prop.** | | **foragers/min.** | |
| contrast | *z* | *p* | *t* | *p* | *z* | *p* | *t* | *p* | *z* | *p* | *t* | *p* |
| *N. tristella* / *Plebeia* sp. | -0.4 | 1 | -0.9 | 1 | -0.8 | 1 | -0.9 | 1 | NA | NA | NA | NA |
| *N. tristella* / *P. occidentalis* | -3 | **0.03** | -3.4 | **0.01** | 0.8 | 1 | 0.2 | 1 | NA | NA | NA | NA |
| *N. tristella* / *Scaptotrigona* sp. 1 | 0.2 | 1 | -0.2 | 1 | -3.5 | **0.01** | -4.2 | **0.003** | NA | NA | NA | NA |
| *N. tristella* / *Scaptotrigona* sp. 2 | 1 | 1 | 0.5 | 1 | -2 | 0.37 | -1.8 | 0.71 | NA | NA | NA | NA |
| *N. tristella / T. ziegleri* | -4.3 | **0.0003** | -6.5 | **<0.0001** | 1.6 | 0.65 | 0.8 | 1 | NA | NA | NA | NA |
| *N. tristella* / *T. angustula* | -4.7 | **<0.0001** | -9.3 | **<0.0001** | NA | NA | NA | NA | NA | NA | NA | NA |
| *Plebeia* sp. / *P. occidentalis* | -2.1 | 0.26 | -2.9 | 0.06 | 1.4 | 0.81 | 1 | 1 | NA | NA | NA | NA |
| *Plebeia* sp. / *Scaptotrigona* sp. 1 | 0.6 | 1 | 0.8 | 1 | -2.4 | 0.18 | -3.1 | **0.05** | NA | NA | NA | NA |
| *Plebeia* sp. / *Scaptotrigona* sp. 2 | 1.2 | 1 | 1.2 | 1 | -1 | 1 | -1 | 1 | NA | NA | NA | NA |
| *Plebeia* sp. / *T. ziegleri* | -3.3 | **0.01** | -5.5 | **<0.0001** | 2 | 0.37 | 1.4 | 1 | NA | NA | NA | NA |
| *Plebeia* sp. / *T. angustula* | -3.6 | **0.01** | -7.8 | **<0.0001** | NA | NA | NA | NA | NA | NA | NA | NA |
| *P. occidentalis* / *Scaptotrigona* sp. 1 | 4 | **0.001** | 3.4 | **0.01** | -3.5 | **0.01** | -4.4 | **0.002** | 3.1 | **0.02** | 3.8 | **0.003** |
| *P. occidentalis* / *Scaptotrigona* sp. 2 | 2.7 | **0.06** | 3.6 | **0.01** | -2.4 | 0.18 | -1.9 | 0.62 | 2.6 | **0.05** | 3.8 | **0.003** |
| *P. occidentalis* / *T. ziegleri* | -2.4 | 0.15 | -1.5 | 1 | 1 | 1 | 0.6 | 1 | -0.3 | 1 | -0.3 | 1 |
| *P. occidentalis* / *T. angustula* | -3.2 | **0.02** | -2.7 | 0.08 | NA | NA | NA | NA | 1.9 | 0.16 | 1.6 | 0.32 |
| *Scaptotrigona* sp. 1 / *Scaptotrigona* sp. 2 | 1 | 1 | 0.9 | 1 | 2 | 0.37 | 1.7 | 0.78 | -0.3 | 1 | -0.01 | 1 |
| *Scaptotrigona* sp. 1 / *T. ziegleri* | -5.9 | **<0.0001** | -6.5 | **<0.0001** | 3.3 | **0.01** | 4.8 | **0.0005** | -3.2 | **0.02** | -5.7 | **<0.0001** |
| *Scaptotrigona* sp. 1 / *T. angustula* | -6.4 | **<0.0001** | -9.4 | **<0.0001** | NA | NA | NA | NA | -2.6 | **0.05** | -4.4 | **0.001** |
| *Scaptotrigona* sp. 2 / *T. ziegleri* | -3.4 | **0.01** | -6.8 | **<0.0001** | 2.6 | 0.10 | 2.2 | 0.35 | -2.7 | **0.05** | -5.7 | **<0.0001** |
| *Scaptotrigona* sp. 2 / *T. angustula* | -3.6 | **0.01** | -9.9 | **<0.0001** | NA | NA | NA | NA | -2.1 | 0.13 | -4.4 | **0.001** |
| *T. ziegleri* / *T. angustula* | -0.9 | 1 | -1.3 | 1 | NA | NA | NA | NA | 2.8 | **0.05** | 2.5 | 0.06 |

***Table S 4.*** *(cont.)*

|  | total pollen | | | |
| --- | --- | --- | --- | --- |
|  | **prop.** | | **foragers/min.** | |
| contrast | *z* | *p* | *t* | *p* |
| *N. tristella* / *Plebeia* sp. | -0.01 | 1 | -1.3 | 1 |
| *N. tristella* / *P. occidentalis* | 1.1 | 1 | -0.2 | 1 |
| *N. tristella* / *Scaptotrigona* sp. 1 | -3.7 | **0.003** | -3.3 | **0.03** |
| *N. tristella* / *Scaptotrigona* sp. 2 | -2.9 | **0.05** | -2.3 | 0.42 |
| *N. tristella* / *T. ziegleri* | 2 | 0.53 | 0.2 | 1 |
| *N. tristella* / *T. angustula* | 1.2 | 1 | -0.6 | 1 |
| *Plebeia* sp. / *P. occidentalis* | 1 | 1 | 1.1 | 1 |
| *Plebeia* sp. / *Scaptotrigona* sp. 1 | -2.9 | **0.05** | -1.8 | 0.92 |
| *Plebeia* sp. / *Scaptotrigona* sp. 2 | -2.4 | 0.19 | -1 | 1 |
| *Plebeia* sp. / *T. ziegleri* | 1.7 | 0.99 | 1.6 | 1 |
| *Plebeia* sp. / *T. angustula* | 1 | 1 | 1 | 1 |
| *P. occidentalis* / *Scaptotrigona* sp. 1 | -4.6 | **<0.0001** | -3.1 | 0.06 |
| *P. occidentalis* / *Scaptotrigona* sp. 2 | -3.9 | **0.002** | -2.1 | 0.62 |
| *P. occidentalis* / *T. ziegleri* | 0.7 | 1 | 0.4 | 1 |
| *P. occidentalis* / *T. angustula* | -0.2 | 1 | -0.3 | 1 |
| *Scaptotrigona* sp. 1 / *Scaptotrigona* sp. 2 | 0.4 | 1 | 0.7 | 1 |
| *Scaptotrigona* sp. 1 / *T. ziegleri* | 6.1 | **<0.0001** | 3.9 | **0.01** |
| *Scaptotrigona* sp. 1 / *T. angustula* | 6.3 | **<0.0001** | 3.6 | **0.02** |
| *Scaptotrigona* sp. 2 / *T. ziegleri* | 5 | **<0.0001** | 2.7 | 0.19 |
| *Scaptotrigona* sp. 2 / *T. angustula* | 4.8 | **<0.0001** | 2.2 | 0.54 |
| *T. ziegleri / T. angustula* | -1.1 | 1 | -0.9 | 1 |

**Table S 5.** Pairwise comparison between the stingless bee species in the amount of sucrose collected (mg) and intake per minute (calculated with the normalized activity). Shown are t- ratios and p-values obtained with contrasts of estimated marginal means (adjustment method: Holm; emmeans R package). Bold values indicate significant differences between species (p ≤ 0.05).

|  | sucrose collection | | | |
| --- | --- | --- | --- | --- |
|  | **amount** | | **intake per min.** | |
| contrast | *t* | *p* | *t* | *p* |
| *N. tristella* / *Plebeia* sp. | -0.1 | 1 | -2.6 | 0.12 |
| *N. tristella* / *P. occidentalis* | -3.2 | **0.03** | -7.1 | **<0.0001** |
| *N. tristella* / *Scaptotrigona* sp. 1 | 1.6 | 1 | -1 | 1 |
| *N. tristella* / *Scaptotrigona* sp. 2 | 0.5 | 1 | -2 | 0.45 |
| *N. tristella* / *T. ziegleri* | 1.3 | 1 | -2.3 | 0.23 |
| *N. tristella* / *T. angustula* | 3.7 | **0.004** | 1.6 | 0.82 |
| *Plebeia* sp. / *P. occidentalis* | -2.9 | 0.06 | -3.7 | **0.004** |
| *Plebeia* sp. / *Scaptotrigona* sp. 1 | 1.7 | 0.97 | 1.6 | 0.82 |
| *Plebeia* sp. / *Scaptotrigona* sp. 2 | 0.5 | 1 | -0.1 | 1 |
| *Plebeia* sp. / *T. ziegleri* | 1.4 | 1 | 0.2 | 1 |
| *Plebeia* sp. / *T. angustula* | 3.7 | **0.004** | 4.5 | **0.0002** |
| *P. occidentalis* / *Scaptotrigona* sp. 1 | 5.0 | **<0.0001** | 5.9 | **<0.0001** |
| *P. occidentalis* / *Scaptotrigona* sp. 2 | 2.6 | 0.15 | 2.4 | 0.19 |
| *P. occidentalis* / *T. ziegleri* | 4.2 | **0.001** | 3.9 | **0.002** |
| *P. occidentalis* / *T. angustula* | 7.8 | **<0.0001** | 10.7 | **<0.0001** |
| *Scaptotrigona* sp. 1 / *Scaptotrigona* sp. 2 | -0.6 | 1 | -1.3 | 1 |
| *Scaptotrigona* sp. 1 / *T. ziegleri* | -0.05 | 1 | -1.4 | 1 |
| *Scaptotrigona* sp. 1 / *T. angustula* | 2.0 | 0.54 | 2.8 | 0.07 |
| *Scaptotrigona* sp. 2 / *T. ziegleri* | 0.6 | 1 | 0.3 | 1 |
| *Scaptotrigona* sp. 2 / *T. angustula* | 2.0 | 0.57 | 3.1 | **0.03** |
| *T. ziegleri* / *T. angustula* | 1.8 | 0.77 | 4.1 | **0.001** |

**Table S 6.** Correlation between resin workforce with pollen or nectar workforce based on Spearman correlation tests conducted for all stingless bee species. Shown are Spearman’s correlation coefficient (rho), S and p-values. Bold values indicate significant differences between treatments (p ≤ 0 .05) after p-value adjustment.

|  | resin/min. correlation with: | | | | | |
| --- | --- | --- | --- | --- | --- | --- |
|  | **pollen/min.** | | | **nectar/min.** | | |
| species | *Spearman’s rho* | *S* | *p* | *Spearman’s rho* | *S* | *p* |
| *N. tristella* | 0.4 | 11.1 | 0.45 | -0.8 | 35.7 | 0.12 |
| *Plebeia* sp. | 0.8 | 3.6 | 0.09 | -0.4 | 27.2 | 0.55 |
| *P. occidentalis* | 0.05 | 19.0 | 0.93 | -0.4 | 28.0 | 0.50 |
| *Scaptotrigona* sp. 1 | 0.5 | 85.0 | 0.16 | 0.2 | 127.6 | 0.53 |
| *Scaptotrigona* sp. 2 | 0.4 | 12.9 | 0.56 | 0.0 | 20.0 | 1.00 |
| *T. ziegleri* | 0.3 | 109.3 | 0.34 | -0.2 | 199.9 | 0.56 |
| *T. angustula* | -0.3 | 711.1 | 0.33 | -0.2 | 199.9 | 0.56 |

**Table S 7.** *Mean percentage of the top five amino acids responsible for differentiating the pollen samples from high and resin collectors according Breiman's random forest algorithm and Mean Decrease Accuracy Indexes.*

|  | Resin collection | |
| --- | --- | --- |
| Amino acids | **Low**  (mean % ± *SD*) | **High**  (mean % ± *SD*) |
| glutamic acid | 9.7 ± 3 | 7.9 ± 2.7 |
| proline | 4.5 ± 2 | 5 ± 2 |
| alanine | 7.8 ± 2.4 | 7.1 ± 1.3 |
| isoleucine | 2.5 ± 0.8 | 1.9 ± 0.9 |
| histidine | 2.1 ± 0.7 | 1.7 ± 0.9 |

# Figures


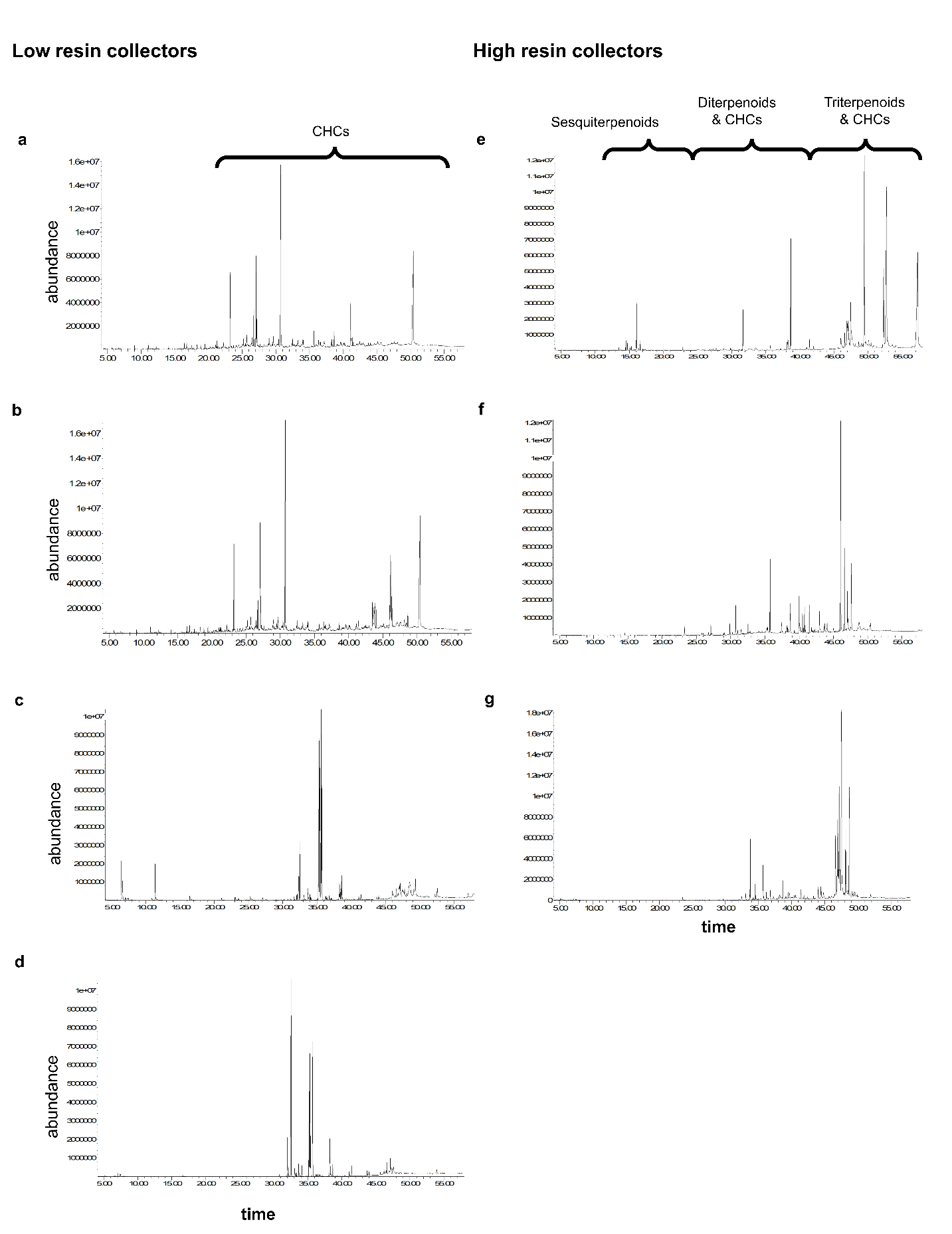


**Figure S 1**. Examples of cuticular profiles (analyzed by gas chromatography coupled with mass spectrometry) of low (left) and high (right) resin collectors. Brackets indicate retention time (RT) regions where specific compound groups elute. Terpenoids are derived from resin and typical for high resin collectors, while cuticular hydrocarbons (CHCs) comprise compounds (e.g., alkanes, alkenes, esters etc.) produced by the bees themselves. (a) profile of Nannotrigona tristella (nest BN9). (b) Plebeia sp. profile (nest BN8). (c) Scaptotrigona sp. 1 profile (nest BN4). (d) Scaptotrigona sp. 2 profile (nest BN2). (e) Ptilotrigona occidentalis profile (nest BN1). (f) Tetragona ziegleri profile (nest BT7). (g) Tetragonisca angustula profile (nest BN3).


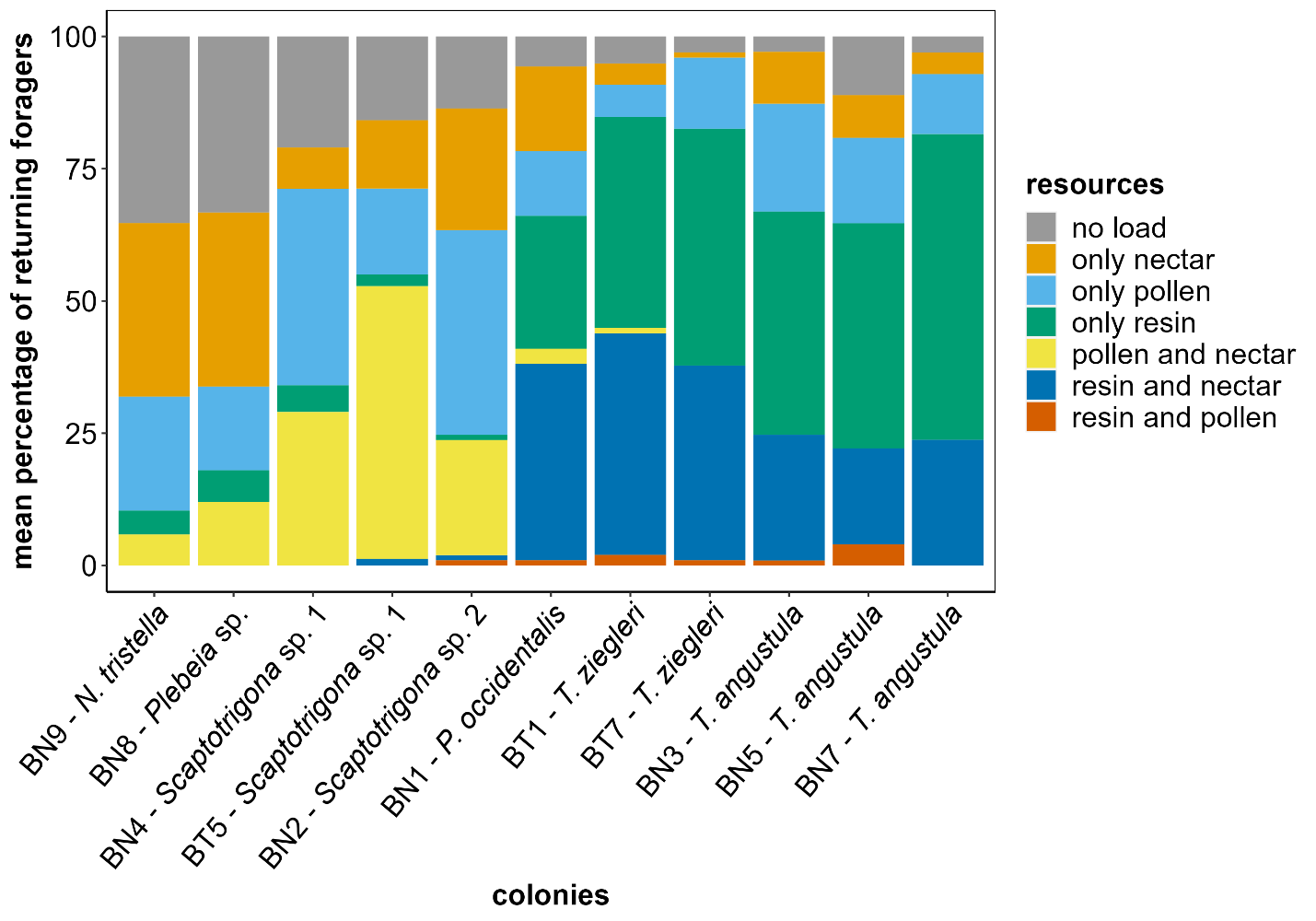


**Figure S 2.** Mean percentage of returning foragers with no load, only nectar, only pollen, only resin, pollen and nectar, resin and nectar, and resin and pollen of the eleven different stingless bee colonies (seven different species) observed in the forest of the Río Canandé and Tesoro Escondido reserves. Colony ID and species name is given.


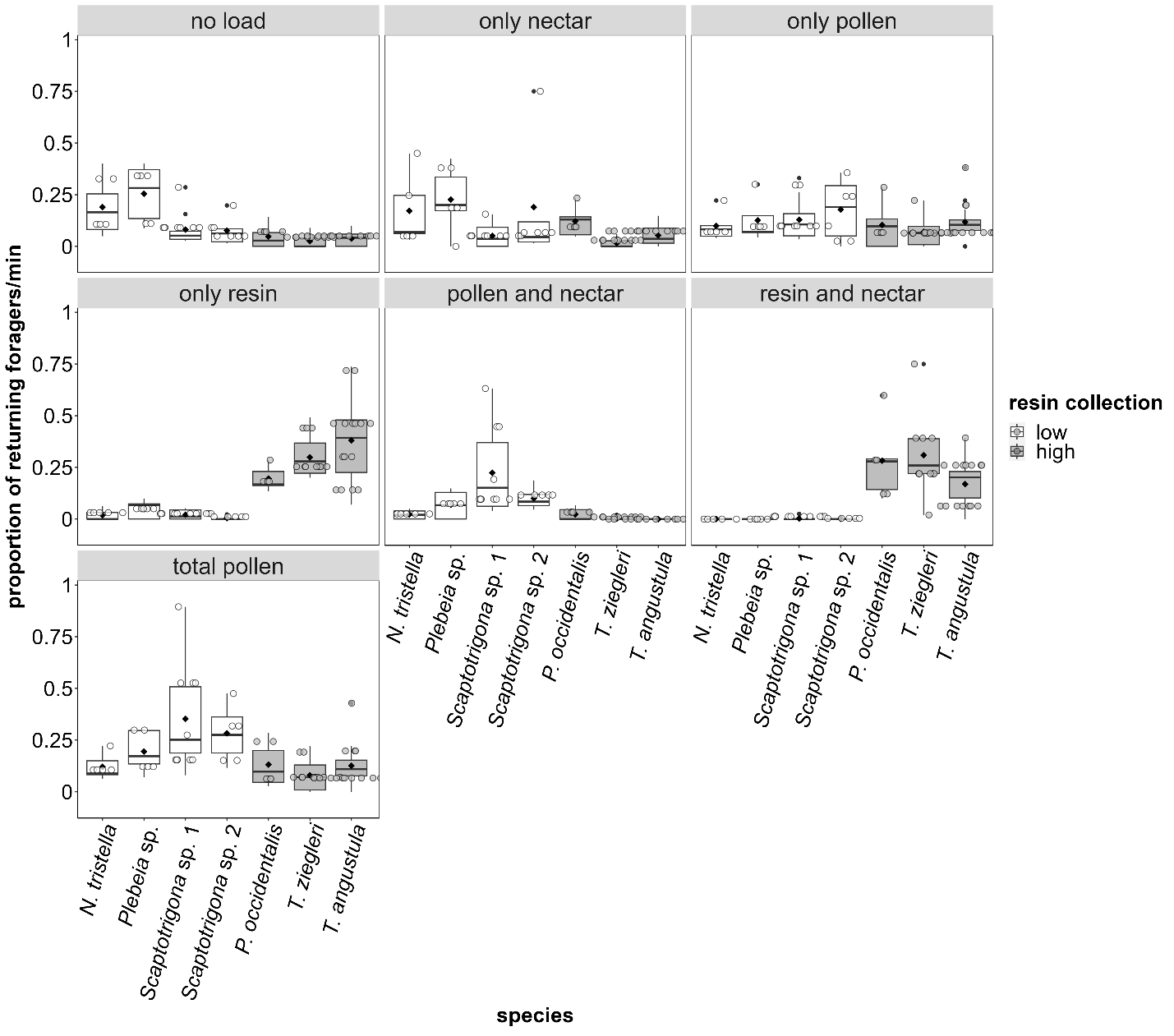


**Figure S 3.** Returning foragers per minute (calculated with the normalized flight activity) with no load, only nectar, only pollen, only resin, pollen and nectar, resin and nectar, and total pollen (sum of only pollen, pollen and nectar and resin and pollen_)_ of the seven different stingless bee species observed in the forest of the reserves. The species are grouped into low (white boxplots) and high (gray boxplots) resin collectors. Box plots display the median (thick bar), lower and upper quartile (boxes), and minimum and maximum values (whiskers) of the data set. The black diamonds represent the mean value of the data set. n = 5, except for Scaptotrigona sp. 1 and T. ziegleri with n = 10, and T. angustula with n = 15.


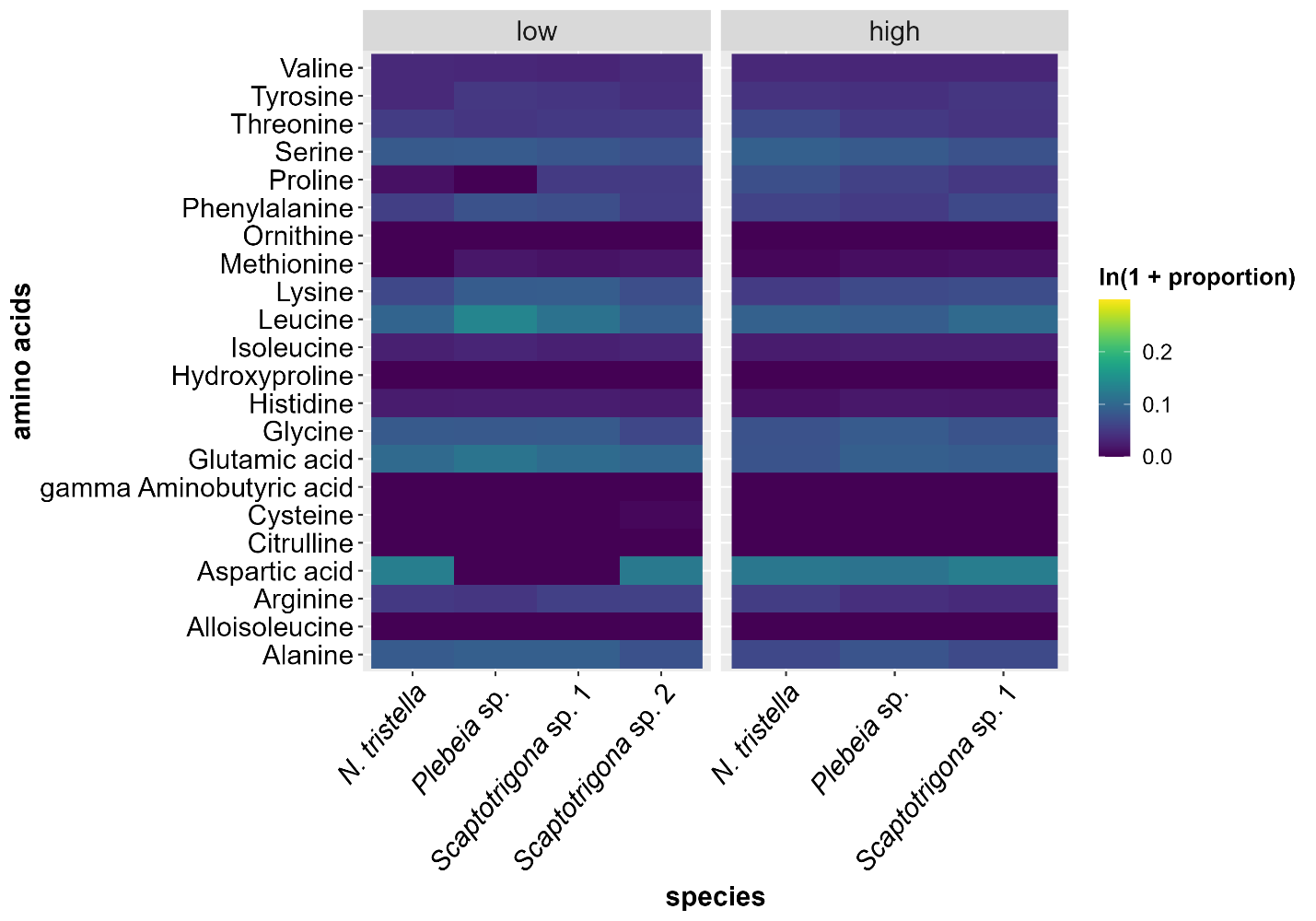


**Figure S 4.** A heat map with the proportion of pollen amino acids in pollen collected by the seven studied stingless bee species. The color scale ranges from 0 to 0.5, where blue indicates that low proportions of those amino acids were found in pollen and yellow indicates that those amino acids were found at high proportions. The heat map was calculated with the natural logarithm of 1 plus the proportion of each amino acid to better display color differences. n = 3, except for N. tristella with n = 2, Scaptotrigona sp. 1 with n = 5, and T. angustula with n = 8.


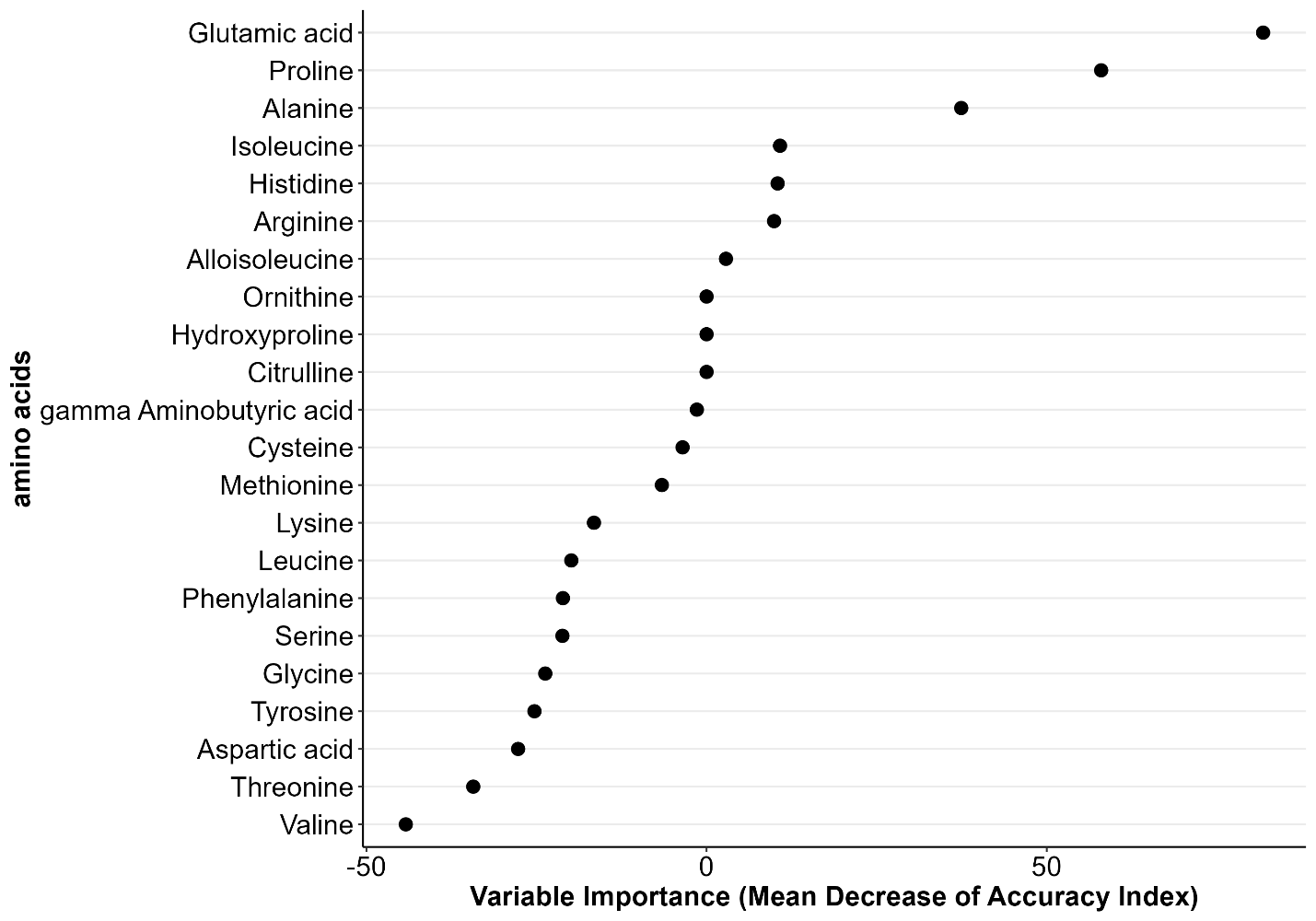


**Figure S 5.** Importance of each pollen amino acid responsible for the cluster between high and low resin collectors showed by the Mean Decrease Accuracy Index (MDAI). It indicates how much the model accuracy decreases when we drop a specific amino acid. The higher the value the more important was this amino acid to differentiate between high and low resin collectors (i.e., the amino acid profile of the pollen of high and low resin collectors differed mainly due to the proportion of those amino acids).

# References

Alencar, A. C., Tölke, E. D., & Mayer, J. L. S. (2020). New perspectives on secretory structures in Clusia (Clusiaceae – Clusiod clade): Production of latex or resins? *Botany*, *98*(3), 161–172. https://doi.org/10.1139/cjb-2019-0103

Amorin, M., de Paula, J. P., da Silva, R. Z., Farago, P. V., & Budel, J. M. (2014). Pharmacobotanical study of the leaf and stem of Mikania lanuginosa for its quality control. *Revista Brasileira de Farmacognosia*, *24*(5), 531–537. https://doi.org/10.1016/j.bjp.2014.10.002

Ankenbrand, M. J., Keller, A., Wolf, M., Schultz, J., & Förster, F. (2015). ITS2 Database V: Twice as Much. *Molecular Biology and Evolution*, *32*(11), 3030–3032. https://doi.org/10.1093/molbev/msv174

Araujo-Murakami, A., & Zenteno Ruiz, F. S. (2006). Bosques de los Andes orientales de Bolivia y sus especies útiles. In M. Moraes, B. Ollgaard, L. P. Kvist, F. Borchsenius, & H. Balslev (Eds.), *Botánica Económica de los Andes Centrales* (pp. 146–161). La Paz, Bolivia: Herbario Nacional de Bolivia, Universidad Mayor de San Andrés, Plural Editores.

Blüthgen, N., Menzel, F., & Blüthgen, N. (2006). Measuring specialization in species interaction networks. *BMC Ecology*, *6*(1), 9. https://doi.org/10.1186/1472-6785-6-9

Burkill, H. M. (1985). *The useful plants of west tropical Africa* (Vol. 4). Royal Botanic Gardens, Kew.

Carlquist, S. (1970). Wood Anatomy of Echium (Boraginaceae). *Aliso: A Journal of Systematic and Floristic Botany*, *7*(2), 183–199. https://doi.org/10.5642/aliso.19700702.07

Curtis, J. D., & Lersten, N. R. (1974). Morphology, Seasonal Variation, and Function of Resin Glands on Buds and Leaves of Populus Deltoides (salicaceae). *American Journal of Botany*, *61*(8), 835–845. https://doi.org/10.1002/j.1537-2197.1974.tb12309.x

Deharo, E., Bourdy, G., Quenevo, C., Muñoz, V., Ruiz, G., & Sauvain, M. (2001). A search for natural bioactive compounds in Bolivia through a multidisciplinary approach. Part V. Evaluation of the antimalarial activity of plants used by the Tacana Indians. *Journal of Ethnopharmacology*, *77*(1), 91–98. https://doi.org/10.1016/S0378-8741(01)00270-7

Dormann, C. F. (2022). *Using bipartite to describe and plot two-mode networks in R*. R package version. Retrieved from https://cloud.r-project.org/web/packages/bipartite/vignettes/Intro2bipartite.pdf

Dormann, C. F., Gruber, B., & Fruend, J. (2008). Introducing the bipartite Package: Analysing Ecological Networks. *R News*, *8/2*, 8–11.

Drescher, N., Klein, A.-M., Schmitt, T., & Leonhardt, S. D. (2019). A clue on bee glue: New insight into the sources and factors driving resin intake in honeybees (Apis mellifera). *PLOS ONE*, *14*(2), e0210594. https://doi.org/10.1371/journal.pone.0210594

Dussourd, D. E., & Denno, R. F. (1991). Deactivation of Plant Defense: Correspondence Between Insect Behavior and Secretory Canal Architecture. *Ecology*, *72*(4), 1383–1396. https://doi.org/10.2307/1941110

Edgar, R. C. (2016a). *SINTAX: A simple non-Bayesian taxonomy classifier for 16S and ITS sequences*. bioRxiv. 074161. https://doi.org/10.1101/074161

Edgar, R. C. (2016b). *UCHIME2: Improved chimera prediction for amplicon sequencing*. bioRxiv. 074252. https://doi.org/10.1101/074252

Edgar, R. C., & Flyvbjerg, H. (2015). Error filtering, pair assembly and error correction for next-generation sequencing reads. *Bioinformatics*, *31*(21), 3476–3482. https://doi.org/10.1093/bioinformatics/btv401

Elliott, B., Wilson, R., Shapcott, A., Keller, A., Newis, R., Cannizzaro, C., … Wallace, H. M. (2021). Pollen diets and niche overlap of honey bees and native bees in protected areas. *Basic and Applied Ecology*, *50*, 169–180. https://doi.org/10.1016/j.baae.2020.12.002

Fernandes, G. W., Oki, Y., Belmiro, M. S., Resende, F. M., Corrêa Junior, A., & de Azevedo, J. L. (2018). Multitrophic interactions among fungal endophytes, bees, and Baccharis dracunculifolia: Resin tapering for propolis production leads to endophyte infection. *Arthropod-Plant Interactions*, *12*(3), 329–337. https://doi.org/10.1007/s11829-018-9597-x

Ferreira, M. E., Cebrián-Torrejón, G., Corrales, A. S., Vera de Bilbao, N., Rolón, M., Gomez, C. V., … Fournet, A. (2011). Zanthoxylum chiloperone leaves extract: First sustainable Chagas disease treatment. *Journal of Ethnopharmacology*, *133*(3), 986–993. https://doi.org/10.1016/j.jep.2010.11.032

Hammel, B. (1989). New Combinations and Taxonomies in Clusiaceae. *Annals of the Missouri Botanical Garden*, *76*(3), 927–929. https://doi.org/10.2307/2399657

Hirschhorn, H. H. (1981). Botanical remedies of south and central America, and the Caribbean: An archival analysis. Part I. *Journal of Ethnopharmacology*, *4*(2), 129–158. https://doi.org/10.1016/0378-8741(81)90032-5

Keller, A., Danner, N., Grimmer, G., Ankenbrand, M., Ohe, K. von der, Ohe, W. von der, … Steffan‐Dewenter, I. (2015). Evaluating multiplexed next-generation sequencing as a method in palynology for mixed pollen samples. *Plant Biology*, *17*(2), 558–566. https://doi.org/10.1111/plb.12251

Keller, A., Hohlfeld, S., Kolter, A., Schultz, J., Gemeinholzer, B., & Ankenbrand, M. J. (2020). BCdatabaser: On-the-fly reference database creation for (meta-)barcoding. *Bioinformatics*, *36*(8), 2630–2631. https://doi.org/10.1093/bioinformatics/btz960

Padovan, A., Keszei, A., Külheim, C., & Foley, W. J. (2014). The evolution of foliar terpene diversity in Myrtaceae. *Phytochemistry Reviews*, *13*(3), 695–716. https://doi.org/10.1007/s11101-013-9331-3

Paniagua-Zambrana, N. Y., Bussmann, R. W., & Romero, C. (2020). Cordia alliodora (Ruiz & Pav.) Oken Cordia lutea Lam. Boraginaceae. In N. Y. Paniagua-Zambrana & R. W. Bussmann (Eds.), *Ethnobotany of the Andes* (pp. 1–6). Cham: Springer International Publishing. https://doi.org/10.1007/978-3-319-77093-2_78-1

Ratnasingham, S., & Hebert, P. D. N. (2007). bold: The Barcode of Life Data System (http://www.barcodinglife.org). *Molecular Ecology Notes*, *7*(3), 355–364. https://doi.org/10.1111/j.1471-8286.2007.01678.x

Rognes, T., Flouri, T., Nichols, B., Quince, C., & Mahé, F. (2016). VSEARCH: A versatile open source tool for metagenomics. *PeerJ*, *4*, e2584. https://doi.org/10.7717/peerj.2584

Sickel, W., Ankenbrand, M. J., Grimmer, G., Holzschuh, A., Härtel, S., Lanzen, J., … Keller, A. (2015). Increased efficiency in identifying mixed pollen samples by meta-barcoding with a dual-indexing approach. *BMC Ecology*, *15*(1), 20. https://doi.org/10.1186/s12898-015-0051-y

Thadeo, M., Azevedo, A. A., & Meira, R. M. S. A. (2014). Foliar anatomy of neotropical Salicaceae: Potentially useful characters for taxonomy. *Plant Systematics and Evolution*, *300*(9), 2073–2089. https://doi.org/10.1007/s00606-014-1037-5

Vignote Peña, S. (2014). *Principales maderas tropicales utilizadas en España: Características, tecnología y aplicaciones*. UPM: E.T.S.I. Montes, Forestal y del Medio Natural (UPM). Retrieved from https://oa.upm.es/32468/

Villagra, C. A., Meza, A. A., & Urzúa, A. (2014). Differences in arthropods found in flowers versus trapped in plant resins on Haplopappus platylepis Phil. (Asteraceae): Can the plant discriminate between pollinators and herbivores? *Arthropod-Plant Interactions*, *8*(5), 411–419. https://doi.org/10.1007/s11829-014-9328-x

Zoghbi, M. G. B., Andrade, E. H. A., Santos, A. S., Luz, A. I. R., & Maia, J. G. S. (1998). Volatile Constituents of the Resins from Protium subserratum (Engl.) Engl. And Tetragastris panamensis (Engl.) Kuntz. *Journal of Essential Oil Research*, *10*(3), 325–326. https://doi.org/10.1080/10412905.1998.9700910
